# Multidrug-resistant tuberculosis (MDR-TB) disease burden in China: a systematic review and spatio-temporal analysis

**DOI:** 10.1186/s12879-016-2151-5

**Published:** 2017-01-10

**Authors:** Peipei Ding, Xiaowen Li, Zhongwei Jia, Zuhong Lu

**Affiliations:** 1 School of Geography, Beijing Normal University, Beijing, 100875 China; 2 National Institute on Drug Dependence, Peking University Health Science Center, Beijing, 100191 China; 3 Department of Biomedical Engineering, College of Engineering, Peking University, Beijing, 100871 China

**Keywords:** Multidrug-resistant tuberculosis (MDR-TB), Disease burden, Spatio-temporal analysis, Systems analysis, Kriging interpolation

## Abstract

**Background:**

Surveillance data on the proportion of incident TB cases with MDR was limited and there is no systematic study of MDR-TB in China to date. Our aim was to estimate MDR-TB disease burden in 2012 and change trends during 2003–2012 using spatio-temporal systematic analysis.

**Methods:**

We systematically searched Chinese and English databases for primary articles and reviews that contain MDR-TB survey data about China during the period of 2003–2012. We estimated the proportion of incident TB cases with MDR in cities which had no data to report in 2012 by Kriging spatial interpolation analysis. The primary outcomes were the proportion of incident TB cases with MDR at 2012 and the change trend during 2003–2012.

**Results:**

Total 487 articles met the screening criteria, including 450 in Chinese and 37 in English, and have been used in analysis. The proportion of incident TB cases with MDR among all cases in 2012 showed clear geographic differences. From 2003 to 2012, the proportion of incident TB cases with MDR in all, new and previously treated TB cases were higher during 2006–2009 and significantly lower during 2010–2012 in comparison with the period during 2003–2005 (*P* < 0.0167). The estimated median proportion of incident TB cases with MDR among all cases, as well as in new and previously treated cases in 2012 was 12.8% (IQR 9.8–17.3%), 5.4% (4.5–7.3%) and 28.5% (20.5–30.9%) respectively, which led to an estimate of 121,600 (IQR93,000–164,350) MDR-TB cases in China.

**Conclusions:**

This estimate of MDR-TB burden is considerably higher than data reported by the Chinese fifth national tuberculosis epidemiological sampling survey in 2010 but close to the WHO report, which implies that detailed investigations of MDR-TB burden in China is needed. This research provides data to guide public health decisions at various scales; methods described here can be extended to estimate of the other chronic diseases as well.

## Background

Based on data emerging from drug resistance surveys and continuous surveillance among notified TB cases, the Global Tuberculosis Report suggested that, globally, an estimated 3.3% of new TB cases and 20% of previously treated cases were infected with Multidrug-resistant tuberculosis (MDR-TB) in 2014 [1]. An estimated 480,000 people who developed MDR-TB and 190,000 people died of MDR-TB; with more than half of them (54%) occurring in India, China and the Russian Federation. The number of MDR-TB patients in China followed India and ranked second in the world. The report estimated that 5.7% (4.5–7.0%) incident TB cases with MDR among new cases and 26%(22–30%) among previously treated cases in China [1].

The Chinese government has been involved in extensive efforts to prevent and control MDR-TB. In 2006, China initiated the fifth round of the Global Fund tuberculosis (TB) project in Hebei, Jilin, Jiangsu, Yunnan, Guangxi, Sichuan, and Shaanxi province to target at MDR-TB specifically [2]. During 2007–2008, the Ministry of Health organized a nationwide baseline survey for TB drug resistance [3], and the results indicated that the prevalence of MDR-TB was 8.32% (95%CI, 7.13 to 9.70) with 120,000 new MDR-TB cases occurring every year, which estimated about 110,000(95%CI, 97,000 to 130,000) new MDR-TB cases occurring every year, which accounted for about a quarter of the globally total number of new MDR-TB cases in a year. The fifth national tuberculosis epidemiological sampling survey in 2010 suggested that the total proportion of incident TB cases with MDR was 6.8%, with 5.4% of new cases and 15.4% of previously treated cases [4].

MDR-TB patients need at least 24 months of treatment in general and the severe cases may need 36 months of treatment,however, the cure rate is only around 50 to 60% [5–8],which makes the economic burden of MDR-TB as high as 10–100 times comparing with non MDR-TB cases [9, 10]. MDR-TB takes longer to treat with second-line drugs, which are more expensive and have more side-effects [11].

Therefore, to control the proportion of incident TB cases with MDR is a top public health priority in China [12].

However, very few cases were tested for TB sensitivity at present time in China, for example, only 280 cases completed TB sensitivity test in the fifth national tuberculosis epidemiological sampling survey in 2010 [4], indicating the survey may not represent whole MDR-TB epidemic. Another important nationwide baseline survey for TB drug resistance in 2007–2008 has lasted 7 years [3], so reliable estimate of the proportion of incident TB cases with MDR is essential to prevent and control MDR-TB. Spatio-temporal analysis refers to the spatial distribution and changing trends with time of the proportion of incident TB cases with MDR [13]. In this study, we aimed to update the estimate of MDR-TB burden in China by a spatio-temporal systematic analysis across 2003–2012.

## Methods

MDR-TB is defined as TB disease caused by organisms that are resistant to isoniazid and rifampicin, two major first-line anti-TB drugs. We divided MDR-TB case into three groups: all cases, new cases, and previously treated cases. The new case is defined as a patient who has received no or less than 1 month of anti-tuberculosis treatment, the previously treated case is defined as a patient who has been treated for 1 month or longer using anti-tuberculosis medication, and the all case was defined as an MDR-TB case regardless of the treatment history [5].

The proportion of incident TB cases with MDR equaled the number of MDR divided by the total number of culture-positive mycobacterium tuberculosis (MTB), multiplied by 100%.We have calculated it for three groups (all, new, and previously treated cases) based on the articles reports respectively.

### Data source and study design

We systematically searched Chinese CNKI, WANGFANG DATA, VIP databases and English PubMed, and Web of Science databases for primary articles and reviews. The search keywords were ‘*multidrug-resistant tuberculosis*’ or ‘*drug-resistant tuberculosis*’ (Appendix 1). We contacted authors for additional information if the information in the articles was not clearly presented.

MDR-TB burden was analyzed and evaluated by five-steps. First, we acquired and screened the proportion of incident TB cases with MDR at provincial and city level respectively through articles retrieval. Second, we classified the proportion of incident TB cases with MDR into three groups (all cases, new cases and the previously treated cases) at the provincial and city level. Third, we estimated the proportion of incident TB cases with MDR based on provincial level. If this data was absent in certain regions in 2012, we used data from 2011 or 2010 considering of 2–3 years treatment duration for MDR-TB and the proportion was unlikely to change significantly within 2–3 years. Fourth, we estimated the proportion in cities during 2012 using the Kriging spatial interpolation analysis method. The primary outcomes were the proportion of incident TB cases with MDR in 2012 and the change trend during 2003–2012.

### Selection and exclusion criteria

Below we describe the selection criteria for the study. The study area included China’s provinces and cities; we considered studies from Jan. 1, 2003 to Dec. 31, 2012. Second, articles was required to include survey time, area, the number of TB cases which tested for TB resistance, MDR-TB case numbers, resistance test methods and standards of classification. When several surveys were all conducted in same area, we kept only the most informative one.

We excluded the articles which contained duplicate information, or the sample size less than 50. We also excluded the articles describing tests that were carried out for specific populations, such as the elderly, children, prisoners and migrants; the articles describing MDR-TB patient co-infected with other diseases, such as HIV, and diabetes also were also excluded.

Data extracted from each article included: survey area, time of the drug resistance test, number of TB cases which received drug susceptibility testing for isoniazid and rifampicin, the number of MDR-TB cases. MDR-TB data was further classified into all cases, new cases and the previously treated case respectively.

Two investigators were independently responsible for data selection, exclusion, and extraction; if there was disagreement, a discussion to agreement would follow; if this failed, a third person was invited to make the judgment.

### Statistical analysis

There were abundant dataset in the eastern and central areas of China, so we estimated the proportion of incident TB cases with MDR at city level (or scale) which had not been report in 2012 by Kriging spatial interpolation analysis in those areas. Kriging spatial interpolation analysis is a classical geostatistical analysis method and based on rules of space at correlation quantize between the sample points, which we can calculate the proportion of incident TB cases with MDR for no data area by using the known sample points area [14–16], the cross validation method can improve the accuracy of prediction. There are scarce data available in the western area, therefore, the spatial interpolation analysis was conducted only in eastern and central area, and used the provincial data in the western areas to estimate the MDR-TB for nationwide.

Considering the stability of the proportion of incident TB cases with MDR at a short period and the data is rarely in same area in different year during 2003–2012, so we classified the 10 years into three periods: 2003–2005, 2006-2009and 2010–2012. There are two reasons that the years 2005 and 2009 were chosen to define the time periods: in 2002, the largest TB project worldwide was launched by the Chinese government to reinvigorate DOTS, and the government (national, provincial, and rural counties) increased funding on TB control nationwide from 2003 [2, 17]; in 2005, China fulfilled its commitment to the WHO to detecting 70% of all new smear-positive cases and to successfully treat 85% of these cases nationwide; On July. 13, 2009, the Ministry of Health of China and the Bill & Melinda Gates Foundation initiated a TB control project in Beijing [17]. Therefore, the years 2003, 2005 and 2009 indicate meaningful time points for TB prevention and control in China.

Change of the proportion of incident TB cases with MDR in the three periods was examined separately using the multiple chi-square tests using SPSS 22.0 (IBM). The *P*-value has a new criterion in the multiple testing, our article involves three pairs comparisons, so the criterion was 0.05/3 = 0.167, when *P* < 0.167, we could get the conclusion: the differences were significant.

## Results

**Fig. 1:**
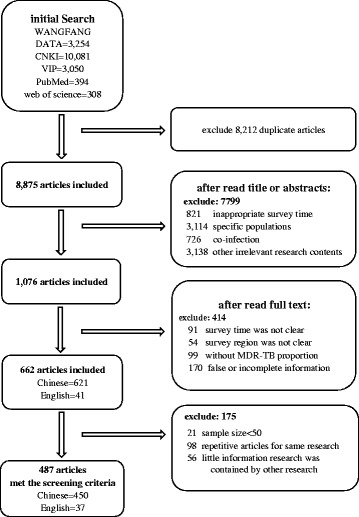
Flow chart of articles selection process in a systematic review of the proportion of incident TB cases with MDR in China

Total 487 articles met the screening criteria, with 450 in Chinese and 37 in English (Fig. 1), among them, 288 were concerned new cases of MDR-TB, 297 reported previously treated cases of MDR-TB and 436 involved all cases of MDR-TB. Table 1 demonstrated the spatial distribution of articles in 31 provinces (see detail distribution of the number of TB and MDR-TB cases in 31 provinces in Appendix 2).

**Table 1:** the number of articles, the proportion of incident TB cases with MDR in 31 provinces

Area	Articles number			the proportion of incident TB cases with MDR (%)		
	all cases	new cases	previously treated cases	all cases	new cases	previously treated cases
Anhui	23	8	8	15.04	4.5	35.61
Beijing	13	10	11	27.82	11.36	35.09
Chekiang	38	31	33	6.6	2.97	28.62
Chongqing	11	7	6	17.72	5.5	26.06
Fujian	15	13	12	9.77	4.63	19.69
Gansu	1	1	1	4.2	3.52	4.67
Guangdong	29	24	24	9.31	6.07	31.11
Guangxi	15	13	14	10.8	4.63	25.53
Guizhou	15	7	8	15.64	4.64	31.14
Hainan	1	1	1	13.49	5.1	29.91
Hebei	15	11	10	12.34	5.33	36.37
Heilongjiang	10	8	7	15.1	6.96	30.2
Henan	27	16	16	11.08	4.03	19.45
Hubei	18	14	17	24.27	4.47	22.37
Hunan	16	10	9	10.59	4.75	28.87
Jiangsu	30	22	22	16.92	5.5	29.3
Jiangxi	8	4	5	17.36	9.35	21.16
Jilin	13	5	6	18.59	2.94	13.71
Liaoning	11	10	9	17.41	6.52	29.36
Mongolia	4	2	2	29.03	7.38	39.71
Ningxia	7	4	4	9.56	7.69	28.4
Qinghai	0	0	0	nodata	nodata	nodata
Shaanxi	10	4	6	12.02	7.74	12.09
Shandong	22	12	12	12.99	5.73	32.64
Shanghai	21	11	13	7.1	3.38	20.28
Shanxi	8	5	5	12.68	4.43	43.02
Sichuan	18	9	10	8.47	4.62	28.22
Tianjin	7	6	6	13.64	9.09	22.73
Tibet	1	1	1	29.8	20.69	56.6
Xinjiang	20	12	12	8.63	7.21	11.82
Yunnan	9	7	7	10	12	14

The median of estimated the proportion of incident TB cases with MDR among all cases, new cases and previously treated cases in 2012 was 12.8% (IQR 9.8–17.3%), 5.4% (4.5–7.3%) and 28.5% (20.5–30.9%) respectively, the number of TB cases was 0.95million in 2012 according to the data from The Data-center of China Public Health Science (Chinese Center for Disease Control and Prevention, CDC), which led to an estimate of 121,600 (IQR93,000–164,350) MDR-TB cases in China.

**Fig. 2:**
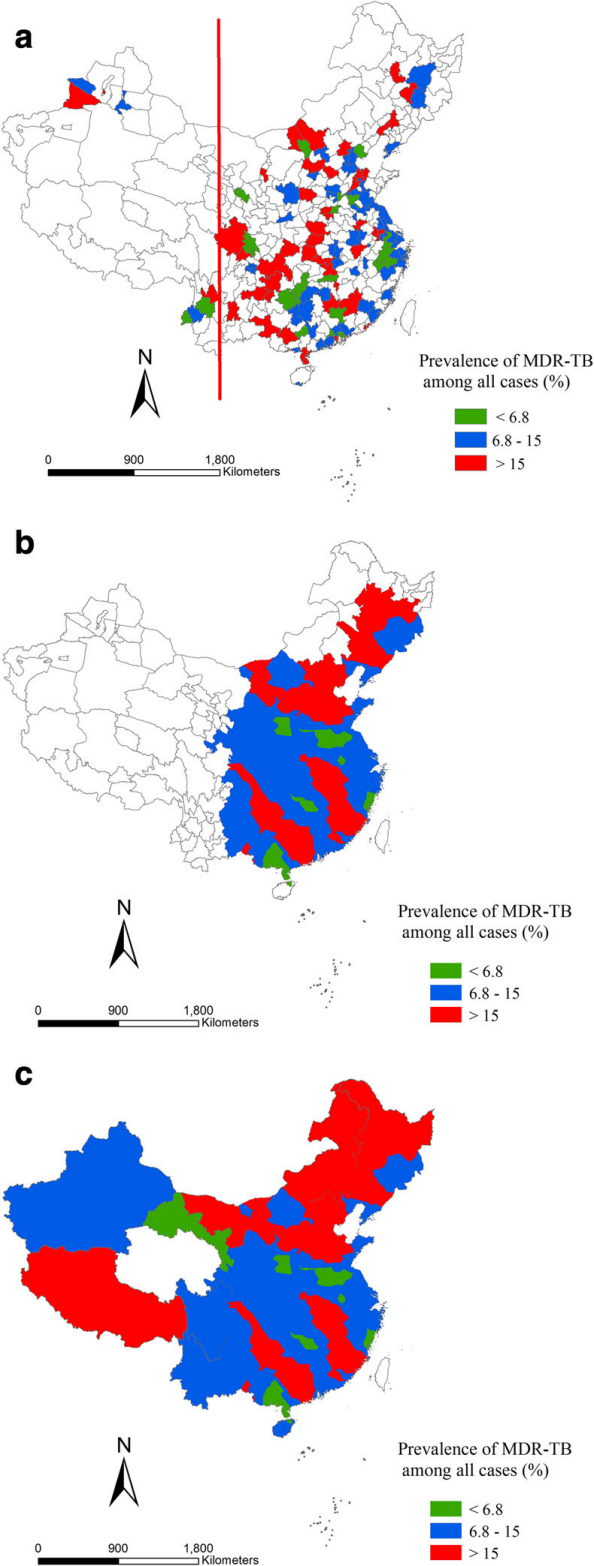
Proportion of incident TB cases with MDR among all cases based on city level. **a** Proportion of incident TB cases with MDR obtained from articles reports, the white blank areas refer to no data available. **b** Kriging interpolation result. We did the Kriging interpolation analysis only in eastern and central area (right side of red line in Fig. 2a) since fewer data was available in western areas. **c** Estimate the proportion of incident TB cases with MDR in nationwide. We used the provincial date in Fig. 3 to make up the west blank area to estimate the proportion of incident TB cases with MDR in nationwide. Therefore, the proportion of incident TB cases with MDR of western region in Fig. 2c was the provincial data from Fig. 3a, and the proportion of incident TB cases with MDR of eastern and central area in Fig. 2c was the Kriging interpolation result from Fig. 2b

**Fig. 3:**
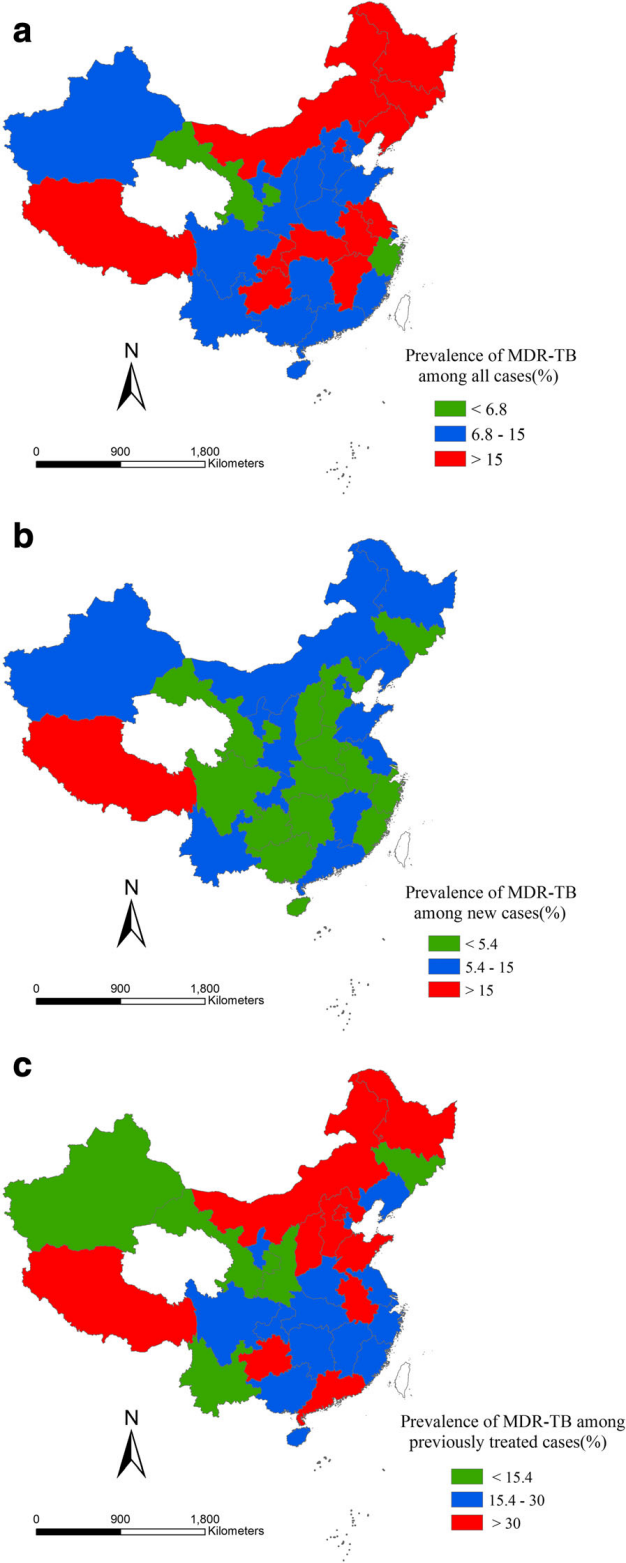
Estimate of the proportion of incident TB cases with MDR based on provincial level. **a** Proportion of incident TB cases with MDR among all cases. **b** Proportion of incident TB cases with MDR among new cases. **c** Proportion of incident TB cases with MDR among previously treated cases

Figure 2a, b and c showed the proportion of incident TB cases with MDR among all cases based on city level (scale); among them, Fig. 2a showed the proportion of incident TB cases with MDR obtained from articles reports, the white blank areas refer to no data available; Fig. 2b presented the Kriging interpolation results. We used Ordinary Kriging method and selected Exponential Function as covariance model according the characteristics of data and this model have a higher accuracy after we tried other models, the case of 127(59%) areas were used to estimate the overall numbers of cases. We did the Kriging interpolation analysis only in eastern and central area (right side of red line in Fig. 2a) since fewer dataset was available in western areas; Fig. 2c showed that the proportion of incident TB cases with MDR in nationwide, We used the provincial data in Fig. 3 to make up the west blank area to estimate the proportion of incident TB cases with MDR in nationwide. Therefore, the proportion of incident TB cases with MDR of western regions in Fig. 2c was the provincial data from Fig. 3a, and the proportion of incident TB cases with MDR of eastern and central area in Fig. 2c was the Kriging interpolation result from Fig. 2b. All spatial analysis was run in ArcGIS 10.2 (ESRI).

**Fig. 4:**
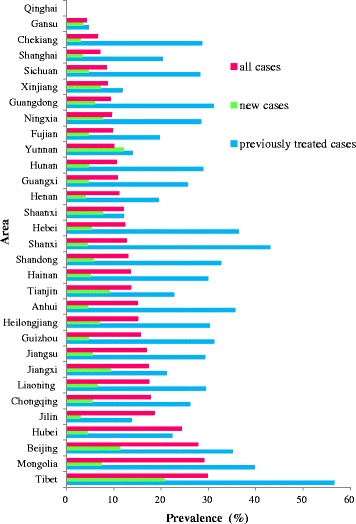
Proportion of incident TB cases with MDR in 31 provinces among all cases, new cases, previously treated cases

There was a clearly geographic difference of the proportion of incident TB cases with MDR among all cases in 2012 at provincial level (scale), with 4.2–6.8% in 2 provinces, 6.8–15% in other 16 provinces and 15–30% in 12 provinces (Fig. 3a). For the new cases, the proportion was 3–5.4% in 15 provinces, 5.4–15% in 14 provinces and 15–21% in 1 province (Fig. 3b). For the previously treated cases, 4.6–15.4% in 5 provinces, 15.4–30% in 15 provinces and 30–57% in 10 provinces (Fig. 3c). It is unclear whether Tibet should be included in the high burden region for new, previously treated and all cases, as only one article report in Tibet was found, which included 198 cases (see detail in Fig. 4, Table 1).

**Fig. 5:**
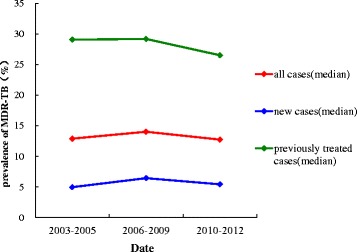
Change trend of the proportion of incident TB cases with MDR. The blue line showed the change trend among new cases in three periods (2003–2005, 2006–2009, 2010–2012); The green line showed the change trend among previously treated TB cases in three periods; The red line showed the change trend among all cases in three periods

During the period from 2003 to 2012, the proportion of incident TB cases with MDR among all, new and previously treated TB cases were all higher during 2006–2009 period and significantly lower during 2010–2012 period compared with 2003–2005 (*P* < 0.167), the proportion of incident TB cases with MDR among previously treated cases was statistically significant higher than that of new cases (*P* < 0.167). (Fig. 5, Tables 2 and 3).

**Table 2:** The number of articles, TB cases, MDR-TB cases, the proportion of incident TB cases with MDR in three periods

Date	all cases					new cases					previously treated cases				
	Article number	TB cases	MDR cases	Proportion (%)	IQR (%)	Article number	TB cases	MDR cases	Proportion (%)	IQR (%)	Article number	TB cases	MDR cases	Proportion (%)	IQR (%)
2003–2005	92	61,363	7,846	12.89	9.21–19.5	70	36,707	2,039	4.98	3.6–8.64	65	10,983	3,001	29.06	20.2–42.65
2006–2009	181	129,058	18,807	14.02	11.23–19.78	105	42,743	3,056	6.45	5.18–10.58	105	16,875	5,022	29.17	25.24–37.02
2010–2012	167	132,929	16,725	12.74	9.6–18.88	112	67,927	3,348	5.44	4.41–7.72	124	23,304	5,783	26.51	19.6–30.21

**Table 3:** The chi square test results of three periods in three groups

Date	All cases		New cases		The previously treated cases	
	*χ* ^2^	*P* value	*χ* ^2^	*P* value	*χ* ^2^	*P* value
2003–2005 compared with 2006–2009	110.242	0.000	83.841	0.000	19.252	0.000
2006–2009 compared with 2010–2012	221.338	0.000	237.354	0.000	121.712	0.000
2003–2005 compared with 2010–2012	1.586	0.208	19.121	0.000	24.652	0.000

**Fig. 6:**
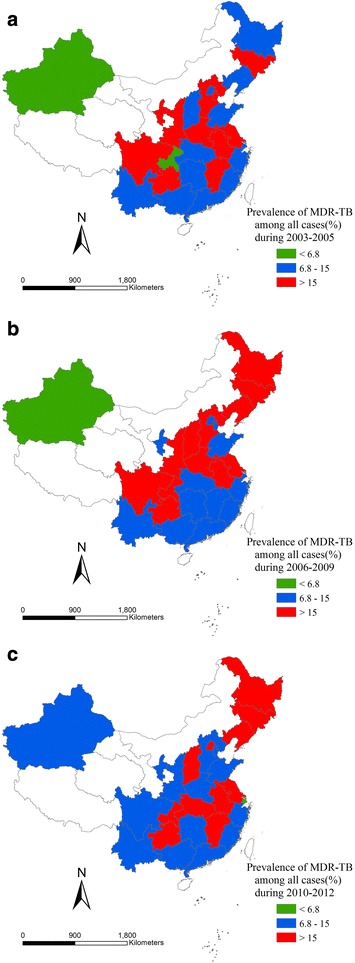
Proportion of incident TB cases with MDR among all cases in three periods. **a** Proportion of incident TB cases with MDR among all cases in 2003–2005. **b** Proportion of incident TB cases with MDR among all cases in 2006–2009. **c** Proportion of incident TB cases with MDR among all cases in 2010–2012

**Fig. 7:**
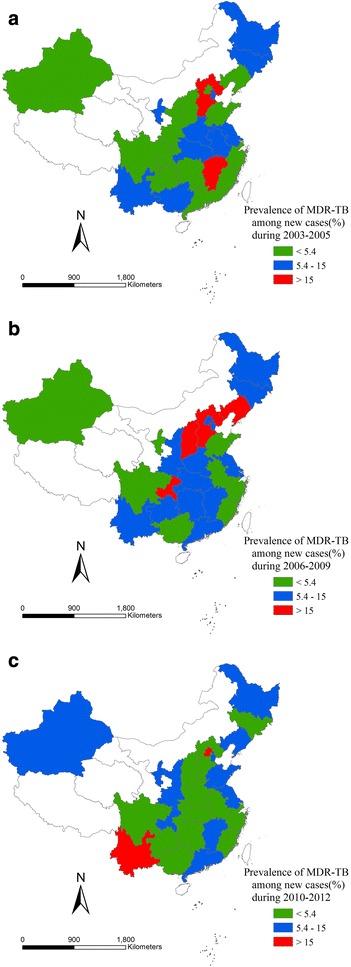
Proportion of incident TB cases with MDR among new cases in three periods. **a** Proportion of incident TB cases with MDR among new cases in 2003–2005. **b** Proportion of incident TB cases with MDR among new cases in 2006–2009. **c** Proportion of incident TB cases with MDR among new cases in 2010–2012

**Fig. 8:**
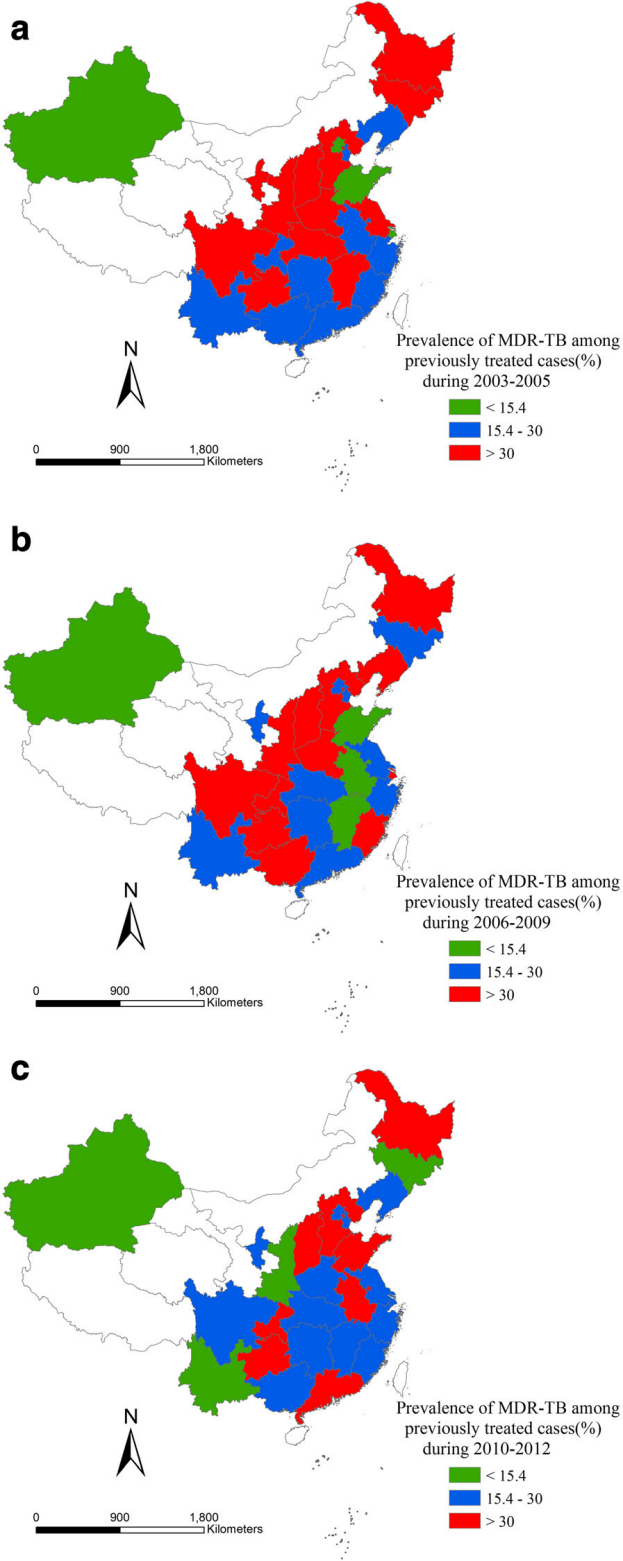
Proportion of incident TB cases with MDR among previously treated cases in three periods. **a** Proportion of incident TB cases with MDR among previously treated cases in 2003–2005. **b** Proportion of incident TB cases with MDR among previously treated cases in 2006–2009. **c** Proportion of incident TB cases with MDR among previously treated cases in 2010–2012

Complete dataset available only on 26 provinces out of 31 provinces around the country; and those datasets were used to represent nationwide data. The spatial distribution maps concerning the three groups in three periods are presented in Figs. 6, 7, and 8.

## Discussion and conclusions

The study results indicate that the proportion of incident TB cases with MDR in China is higher than other international regions [1]. Factors contributing to the high burden may include economic development, poor knowledge and side effects of TB treatment, poor quality DOTS, lack of coordination of medical services, unsatisfactory supervision of treatment and poor infection control [18–21].

The estimates results in our study are higher than the results in the fifth national tuberculosis epidemiological sampling survey in 2010 [4] (12.8vs.6.8% in all cases, 5.4 vs.5.4% in new TB cases; 28.5 vs.15.4% in the previously treated cases). The difference may be due to small sampling size in the sample survey which only provided susceptible test for 280 TB cases, including 241 new cases and 39 previously treated cases.

While, our estimated the proportion of incident TB cases with MDR among new cases and previously treated cases were close to the result from Global tuberculosis Report in 2013, 5.4% (4.5–7.3%) vs. 5.7% (4.5–7.0%) in new TB cases; 28.5% (20.5–30.9%) vs.26% (22–30%) in the previously treated cases. There was no information concerning the proportion of incident TB cases with MDR among all cases in Global tuberculosis Report [1].

It is not a surprise that, our study reveals substantial spatial differences in MDR-TB burden, consistent with previous work [17]. We believe that the difference might be linked to factors including local economy, education, population density and mobility, ethnic minority populations, and distribution of relevant diseases (such as HIV/AIDS) based on geographic features. The high burden provinces, Guangdong, Anhui, Jiangsu and Hebei are good examples for relevant disease of AIDS and density of population [22]. Guangdong has much more complicated factors, such as large migrant population [23].

Our study also indicates that the proportion of incident TB cases with MDR among all TB cases had a significantly higher during 2006–2009 periods and then showed a decreased change during 2010–2012 compared with 2003–2005. The high rate of discovery of TB cases stimulated by the TB project worldwide and the Chinese government’s commitment to detecting 70% of all new smear-positive cases and successfully treat 85% of these cases nationwide contribute to the increase of proportion during 2006–2009. This increase is clearly resulted from the significant efforts of the Chinese government to prevent and control TB by launching a series of policies in past decades, and not a real aggravation of MDR-TB burden in China [17]. Following the implementation of policies concerning TB prevention and control, a decline of the proportion of incident TB cases with MDR would be expected.

The proportion of incident TB cases with MDR differed significantly among the three groups. The new case group was an epidemiological index which better reflect the effectiveness of TB control in recent times. The previously treated case group was a mixed index which is a reflection of both TB control conditions and treatment efficacy in the past [24]. According our estimates, in the past 10 years, our research results show that the proportion of incident TB cases with MDR among new cases has only little change, among previously treated case was shown to be on the decline, but the proportion was extremely higher than that of new cases. Therefore, we should strengthen the drug management of MDR-TB patients, and programmatic management of multidrug-resistant tuberculosis (MDR-TB), including standardized high quality DST for patients, especially in high MDR-TB burden regions [25].

Our study has several strengths. First, the search strategy covered several databases and reported in two languages which involved all provinces except for Qinghai; in addition data for Hong Kong, Taiwan and Macao were also unavailable, which reveals national MDR-TB burden. Second, we propose a spatial systematic analysis by using Kriging’s interpolation method which relies on available data and predict the absence of data from some cities in China. Such analysis would improve local estimates as well as reduce uncertainty in regional and national estimates. The national quantitative map presented here illustrates distribution of the proportion of incident TB cases with MDR. This study is the first attempt to quantify the national burden of MDR-TB in China.

Although we used the strict search strategy and quality control as soon as possible, there still maybe some potential (major) source of bias in this article. First, the DOTS strategy has a significant impact on the prevalence of MDR-TB, although China has realized 100% complete coverage for DOTS strategy in 2007 [26], the implement quality of DOTS strategy have differences in different areas of China, especially in the poor economic areas [27, 28]; Second, since eligible published reports were not available for all of the 31 provinces/municipalities, and not every study explored MDR-TB in all three groups and three periods, therefore, we only carried out change trend analysis in the data rich area and spatial interpolation analysis in central and eastern regions based on city scale; Third, due to the geographical, historical and cultural factors [29, 30], few articles reports about the proportion of TB cases with MDR in the Western areas of China, and the missing data of western regions may affect the reliability of our study result, therefore, we have to use provincial centers data to estimate the entire province based on there was no other data sources in these areas; Without long-term nationwide surveillance data, the results obtained here are so far the best data source to show the proportion of incident TB cases with MDR among China in the past 10 years.

These bias will hopefully encourage other investigators to conduct further prospective studies, which include the collection of high quality epidemiological and microbiological surveillance data, the monitoring of change trends of the proportion of incident TB cases with MDR, and the detection of key groups of people for further research and improved TB control in the future [21].

Our research concerning the proportion of incident TB cases with MDR provides an initial assessment of the MDR-TB disease burden in China. Although research is limited by the data source, it sheds light on the prevalence and change trends of the proportion in China. Detailed investigations of MDR-TB burden are needed in China; this research provides a valuable tool to guide public health decisions at various scales and can be extended to estimates of the other chronic diseases.

## Appendix 1

**Search strategy**

We applied the PRISMA guidelines for this systematic review.

We systematically searched Chinese CNKI, WANGFANG DATA, VIP databases and English PubMed, and Web of Science databases for primary articles and reviews. The search keyword was ‘multidrug-resistant tuberculosis’ or ‘drug-resistant tuberculosis’. In fact, in the process of search, we search all articles contains ‘multidrug-resistant tuberculosis’ or ‘drug-resistant tuberculosis’ in the full text to avoid miss articles. Articles were also searched manually and, if required and when it was feasible, authors were contacted directly for unpublished data and additional information.

## Appendix 2

**Table 4:** Distribution of the number of cases

Areas	date	TB Case			MDR-TB Case		
		all	new	previously treated	all	new	previously treated
Anhui	2012	7,388	2,599	994	1,111	117	354
Beijing	2012	417	88	57	116	10	20
Chekiang	2012	11,216	9,439	1,887	740	280	540
Chongqing	2012	6,054	2,091	495	1,073	115	129
Fujian	2012	4,462	1,834	523	436	85	103
Gansu	2012	834	341	493	35	12	23
Guangdong	2012	7,033	4,758	1,305	655	289	406
Guangxi	2012	3,111	2,181	936	336	101	239
Guizhou	2012	2,481	474	334	388	22	104
Hainan	2012	126	1,196	341	17	61	102
Hebei	2012	3,971	2,927	866	490	156	315
Heilongjiang	2012	1,437	819	351	217	57	106
Henan	2012	3,502	1,813	622	388	73	121
Hubei	2012	5,938	2,060	2,097	1,441	92	469
Hunan	2012	1,180	547	239	125	26	69
Jilin	2012	1,818	102	890	338	3	122
Jiangsu	2012	9,058	5,531	1,536	1,533	304	450
Jiangxi	2012	1,365	813	189	237	76	40
Liaoning	2012	2,860	1,580	780	498	103	229
Mongolia	2012	2,556	610	1,040	742	45	413
Ningxia	2012	2,092	91	169	200	7	48
Qinghai	2012	nodata	nodata	nodata	nodata	nodata	nodata
Shaanxi	2012	1,165	4,238	513	140	328	62
Shandong	2012	3,379	646	432	439	37	141
Shanghai	2012	13,934	9,702	1,277	990	328	259
Shanxi	2012	891	609	172	113	27	74
Sichuan	2012	2,656	2,012	489	225	93	138
Tianjin	2012	1,393	385	88	190	35	20
Tibet	2012	198	116	53	59	24	30
Xinjiang	2012	1,714	749	533	148	54	63
Yunnan	2012	694	152	36	113	7	5

## Appendix 3

**List of references for the 487 studies**

1. W Li, Z Ma, H Zhang, et al. Epidemic status of MDR-TB of Kaifeng Prefecture in Henan[J]. Henan J Prev Med, 2014, 25(2): 100–117.
2. W Li, X Li, Y Qu, et al. Investigation of multidrug-resistance of smear-positive pulmonary tuberculosis patients in Kaifeng city[J]. Chin. J of PH M., 2012, 28(4): 453–454.
3. J Su, G Liang, L Bao, et al. Analysis of culture and drug-resistance of Mycobacterium tuberculosis in Kunming [J]. Chin J Anti tuberculosis, 2012, 34(1): 32–35.
4. H Qiu, Z yan, T Yin. Monitoring Analysis of drug -resistance TB patients in Kunming city [J]. Soft Science of Heath, 2013, 27(1): 37–38.
5. Z Bi, H Ren, F Gao, et al. Analysis of drug resistance trend on tuberculosis in Lang fang city [J]. Journal of Clinical Pulmonary Medicine, 2012, 17(1): 75–77.
6. L Luo, Z Bi, D Jiang, et al. Tuberculosis drug resistance and major epidemiological characteristics analysis of Langfang city[J]. J Med Pest Control, 2014, 30(11): 1188–1193.
7. Q Zhou, Z F, Z S, et al. Analysis of the drug resistance of clinical isolated mycobacterium tuberculosis [J]. The committee of Zhejiang Laboratory Medicine Academic Annual Meeting. [C]. Zhejiang, 2006: 799–801.
8. Y Guo, Y Zhang, Y Li. Analysis of the drug-resistance of clinical isolated Mycobacterium tuberculosis [J]. Chinese Journal of Health Laboratory Technology, 2006, 16(6): 740.
9. Y Wang, H Jing, J Wang, et al. monitoring Analysis of clinical TB resistance [J]. Shandong Med J, 2011, 51(49): 38–40.
10. C Qi. Analysis of the drug-resistance of Mycobacterium tuberculosis in Linyi city in 2010 [J]. JCM, 2011, 9(12): 16–17.
11. Y Zhao. Drug resistance in patients with pulmonary tuberculosis dynamic monitoring and analysis in Linyi City [J]. China Health Care Nutrition, 2013, (10): 16–17.
12. K Lv, G Liao. Treatment effect of anti-tuberculosis drug-resistance and multi-drug resistance of TB inpatients [J]. Journal of Clinical Pulmonary Medicine, 2006, 11(3): 307–308.
13. H Chang, K Li, T Ma, et al. Characteristics of rpoB and katG gene mutation in drug-resistant Mycobacterium tuberculosis in Lu’an[J]. Journal of Tropical Diseases and Parasitology, 2014, 12(4): 187–193.
14. Y Lin, Y Sun, S Shi. Monitoring Analysis of drug-resistant of culture-positive pulmonary 184 new treated tuberculosis patients with in Longchang county [J]. Medical Information, 2012, 25(6): 102–103.
15. J Wang, B Li, X Sun, et al. monitoring Analysis of drug-resistance TB in Longchuan county [J]. J Med Pest Control, 2012, 28(2): 224–225.
16. X Zhang, T Feng. Research and analysis on the monitoring of clinical TB resistance [J]. J Medical Forum, 2013, 34(4): 59–61.
17. S Zhang, W Xu. Analysis on the drug-resistant of 69 cases with smear-positive pulmonary tuberculosis in Ma’anshan City in 2007[J]. Anhui J Prev Med, 2010, 16(1): 29–30.
18. Z Chen, P Zhang, H Yang, et al. Drug resistance of Mycobacterium tuberculosis in Mianyang[J]. J Pre Med Inf, 2012, 29(2): 133–135.
19. X Zhu, H Wang, F Jin. Analysis of multidrug-resistance Mycobacterium tuberculosis in some areas [J]. Chinese Primary Health Care, 2010, 24(2): 75.
20. Y Xu, Q Ji, Y Yu, et al. Analysis of Anti-TB drug resistant surveillance in a city in 2012[J]. Guide of China Medicine, 2013, 11(31): 370–371.
21. S Liang, F Yang, C Guo. The analysis of the causes of drug-resistant TB in a city[J]. Guide of China Medicine, 2011, 9(31): 257–258.
22. X Cao, G Wang, W Ma. Analysis on the drug resistance of 178 mycobacterium tuberculosis in hospital [J]. J Prev Med Chin PLA, 2015, 33(1): 89.
23. L Chen, M Luo, F Ye. Analysis of anti-TB drugs resistance among in patients in 2005[J]. Journal of Modern Clinical Medicine, 2007, 33(1): 13–14.
24. H Xiao, X Wang, F Wang, et al. Analysis of drug resistance situation and trend on tuberculosis patients in a hospital during 2007–2009[J]. Ningxia Med J, 2010, 32(10): 966–967.
25. L Fan, G Li, J Zhang, et al. Research on the discovery ways of multidrug-resistance pulmonary tuberculosis patients[J]. J Med Pest Control, 2013, 29(12): 1409–1410.
26. W Yuan, H Zhou, M Zhang. Drug-resistance situation and factor of multidrug-resistance tuberculosis patients [J]. Guizhou Medical Journal, 2005, 29(12): 1113–1114.
27. M Tan, X Liu, X Guo, et al. Epidemiological status of multidrug-resistant tuberculosis[J]. Chin J Nosocomiol, 2014, 24(17): 4292–4294.
28. Y Yang, S T, Q Z, et al. The changes and the significance of cellular immune function in peripheral blood of patients with multidrug-resistant and extensively drug-resistant pulmonary tuberculosis [J]. Chin J Tuberc Respir Dis, 2011, 34(2): 109–113.
29. L Zhu. MDR-TB and its prevention analysis [J]. J Medical Forum, 2015, 36(2): 81–82.
30. D Pei, J Wang, Y Wang, et al. Study on drug resistance of multidrug resistant tuberculosis [J]. Laboratory Medicine, 2008, 5(4): 79–80.
31. F Mi, J Du, C Xu, et al. Baseline survey of multidrug-resistant tuberculosis (MDR-TB) in MDR-TB diagnosis and management pilot areas [J]. Chin J Antituberc, 2011, 33(8): 466–470.
32. L Liu, K Luo, T Zhang, et al. Analysis on clinical drug resistance of multi-drug resistant Mycobacterium tuberculosis [J]. Laboratory Medicine, 2010, 25(9): 683–685.
33. L Yin, H Sun, Y Zhou, et al. Evaluation of rifampicin resistance as the screening indicator for multi-drug resistant tuberculosis [J]. Journal of Clinical Pulmonary Medicine, 2013, 18(9): 1656–1658.
34. X Shen, J Mei, M Shen. The effect of drug resistance on treatment of tuberculosis [J]. Journal of the Chinese Anti tuberculosis Association, 2006, 2006, 28(5): 309–312.
35. C Zheng, Z Cui. Prevalence survey and analysis of drug-resistant tuberculosis [J]. Lab Med Clin, 2014, 11(9): 1156–1158.
36. H Li. Study on bacterial types and treatment of drug-resistant TB patients [J]. China Medical Engineering, 2013, 21(6): 162–166.
37. K Qiu, X Shen, D Huang, et al. Analysis of drug-resistant tuberculosis patients [J]. China&Foreign Medical Treatment, 2014, (10): 190–191.
38. Y Zhao. Testing analysis of 159 TB patients with drug-resistance [J]. PJCCPVD, 2010, 18(7): 946.
39. J Shao, X Wang, W Zhang, et al. Drug resistance survey of mycobacterium tuberculosis in Nanjing [J]. Jiangsu J Prev Med, 2012, 23(3): 42–43.
40. Y Lin, D Xu, Z Wang. Analysis on drug resistance of inpatients with tuberculosis in Nanjing [J]. Modern Preventive Medicine, 2008, 32(23): 4686–4690.
41. H Niu, B Zhang, Y Zhou. Study on the drug resistance TB in Nanyang city [J]. Yiayao Qianyan, 2014, (12): 99.
42. Y Su, Y Liu, Y Ma, et al. Test results analysis of TB patients with drug susceptibility in Inner Mongolia Fourth Hospital[A]. The committee of Inner Mongolia Natural and Science Academic Annual Meeting. 2012: 826–827.
43. Y Che, M Yu, G Ping. Analyzing 1149 drug resistant M. tuberculosis [J]. Chinese Journal of Health Laboratory Technology, 2010, 20(6): 1494–1495.
44. Y Che, M Yu, G Ping. Analysis of the status and risk factors of drug resistant tuberculosis in Ningbo [J]. Chinese Journal of Health Laboratory Technology, 2013, 23(2): 495–497.
45. X Wang, F Wang, H Xiao, et al. Study on the drug resistance of mycobacterium tuberculosis in 2012 in Nanjing [J]. Health must-read, 2013, 12(11): 309.
46. X Wang, F Wang, H Xiao, et al. Analysis of the drug susceptibility monitoring results of mycobacterium tuberculosis in Ningxia [J]. Ningxia Med J, 2015, 37(1): 78–80.
47. D Hu, Q Li, Z Wang, et al. Analysis of the drug susceptibility monitoring results of 44 mycobacterium tuberculosis in Pingxiang country [J]. Experimental and Laboratory Medicine, 2010, 28(2): 137–138.
48. L Zhu, Z Feng, H Liu. Analysis of drug resistance status of mycobacterium tuberculosis in Puyang[J]. Chinese Community Doctors, 2014, 30(22): 15–17.
49. Y Zheng, H Deng, J Chen, et al. The study on drug resistance of registered population with active tuberculosis in Puto District during 2000 to 2008[A]. Chinese Antituberculosis Association. Proceedings of the 12th academic conference of Chinese [C]. Linzhi: Chinese Anti tuberculosis Association, 2010: 335–339.
50. Y Wu, C Xu, J Xiao. Drug resistance tuberculosis monitoring analysis in Qiandongnan Prefecture [J]. Yiayao Qianyan, 2014, (17): 263–264.
51. F Wang, Q Zhu, F Fei. Analysis of primary drug resistance of 552 strains of Mycobacterium tuberculosis in Qingpu district [J]. Journal of Clinical Pulmonary Medicine, 2010, 15(6): 810–811.
52. F Wu. Monitoring analysis of drug-resistance TB during 2006–2010 in Qingtongxia city[J]. Baotou Journal of Medicine, 2012, 36(1): 23–24.
53. Y Cheng, X Zeng, Y Wu. Dynamic analysis of the status of drug resistance of Mycobacterium tuberculosis of pulmonary tuberculosis cases from 2001 to 2005 in Quanzhou [J]. Chin J Antitubercul, 2009, 31(2): 68–70.
54. Z Wang, S Chen, Y Cheng. Dynamic observation of drug resistance for tuberculosis in Quanzhou [J]. Journal of Clinical Pulmonary Medicine, 2005, 10(6): 755–756.
55. R Ke, R Zheng, X Zhang, et al. Analysis on epidemic and relevant factors for drug-resistant tuberculosis in Xiamen city[J]. Chin J Antitubercul, 2014, 36(2): 93–97.
56. Y Deng, Y Zhang, J Zheng, et al. Study on molecular characteristics regarding DNA genotype of Mycobacterium tuberculosis clinical strains in Shandong[J]. Chin J Epidemiol, 2010, 31(3): 321–323.
57. H Wang, Y Wang, C Yu, et al. Analysis of drug resistance TB in 13 counties in Shandong [A]. Chinese Medical Association. The proceedings of Chinese Medical Association National TB Conference in 2008[C]. Changsha: Chinese Medical Association, 2008: 258–260.
58. X Guo, J Chi, W Li, et al. Analysis of drug resistance of 1184 cases sputum culture positive TB patients in Shandong Province Thoracic Hospital [A]. Professional committee of combination of traditional Chinese and western medicine breathing disease in Shandong [C]. Jinan: 2011: 201–204.
59. H Xiao, R Wang, T Zhang, et al. Analysis of drug resistance surveillance on tuberculosis in Qinba region of Shanxi [J]. Chin J Pharmacoepidemiology, 2010, 19(10): 555–558.
60. Y Liu, S Tang, Q Zhang, et al. Analysis on first-and stored-line drug resistant patterns in 518 Mycobacterium tuberculosis strains in Shanghai [J]. Chin J Infect Dis, 2011, 29(9): 544–548.
61. J Li. Resistance to second-line drugs in first-line drugs resistance TB patients in Shanghai and the relationship between resistant to second-line drugs and gene loci in Beijing family Mycobacterium Tuberculosis [D]. Shanghai: Fudan University, 2011.
62. Z Zhang, H Xiao. Clinical study on pulmonary tuberculosis with first retreatment in Shanghai Pulmonary Hospital [J]. Chin J Antitubercul, 2010, 32(1): 1–5.
63. J Mei, X Shen, M Shen, et al. Survey of drug-resistant M.tuberculosis in Shanghai, China [J]. Chin J Antitubercul, 2007, 29(5): 395–398.
64. L Tang, H Yan, Y Wu, et al. Analysis of drug resistance mycobacterium tuberculosis during 2008–2010 in Minhang district in Shanghai [J]. Chin J Antituberc, 2014, 26(3): 216–218.
65. X Shen. Study on the drug-resistance TB epidemic rule and the correlation analysis of embB gene mutation in 306 and drug resistance of mycobacterium tuberculosis in Shanghai [D]. Shanghai: Fudan University, 2008.
66. M Chen, Q Qian, W Wang, et al. Analysis of drug resistance tuberculosis in Qingpu district, Shanghai during 2006–2010 [J]. Shanghai Journal of Preventive Medicine, 2011, 23(12): 596–597.
67. Y Fan, H Xiao, J Mei. Analysis of drug resistance to antituberculosis drugs of the first time retreated pulmonary tuberculosis patients in Shanghai [J]. Chin J Tuberc Respir Dis, 2006, 29(10): 698–701.
68. H Wang, W Yang, J He, et al. Tuberculosis bacili culture and the drug susceptibility results analysis in Shaoyang city from during 2006–2008[J]. Practical Preventive Medicine, 2009, 16(4): 1276–1277.
69. H Gao, Q Chen, F Jin, et al. An analysis on the status of drug-resistance of tuberculosis in Shaoxing city [J]. Zhejiang Prev Med, 2014, 26(3): 242–262.
70. F Jin. Study on the drug resistance of 833 mycobacterium tuberculosis in Shaoxing [J]. Modern Journal of Integrated Traditional Chinese and Western Medicine, 2007, 16(10): 1385–1386.
71. J Lv, Y Yang, Q Wu, et al. Comparative analysis of 2005, 2009 tuberculosis drug-resistance surveillance in Shenzhen[J]. Chin J Prim Pharm, 2011, 18(13): 1741–1742.
72. B Huang, Y Qiu, J Xiao, et al. Study on drug resistance of Mycobacterium tuberculosis in Bao’an district [J]. Modern Preventive Medicine, 2006, 33(4): 538–542.
73. D Luo, Q Wu, J Pan, et al. The monitoring of drug resistant of mycobacterium tuberculosis in Shenzhen [J]. Journal of Clinical Pulmonary Medicine, 2006, 11(3): 304–305.
74. L Xu, Y Yang, Q Wu, et al. Analysis of first line anti-TB drug resistance surveillance in Shenzhen [J]. Chin J Antitubercul, 2010, 32(4): 204–207.
75. Y Sun, G Zuo. Analysis of the drug-resistance mycobacterium tuberculosis in Huanggu district in Shenyang [J]. 2003, (49): 120–121.
76. H Shen, Y Hua, S Wang, et al. Drug resistance analysis of clinical isolates of mycobacterium tuberculosis in Shiyan[J]. China Medical Herald, 2007, 4(16): 25.
77. Z Sun, J Liu, C Lv, et al. Study on the drug resistance of 1552 mycobacterium tuberculosis in Shijiazhuang[J]. Clinical Focus, 2007, 22(22):1655–1656.
78. L He, J Zhu, Z Zhao, et al. Analysis on drug-resistant strains of 268 TB patients with initial treatment in Shijiazhuang city [J]. Occup and Health, 2010, 26(1): 67–69.
79. H Yang. Analysis of the drug resistance tuberculosis patients in Shijiazhuang [J]. Chinese Journal of Health Laboratory Technology, 2006, 16(8): 999–1000.
80. L Wang, L Wu, J Zhang, et al. Analysis of drug resistance of patients with tuberculosis after implemention of DOTS strategy in Hefei city [J]. Modern Preventive Medicine, 2012, 39(15): 3951–3955.
81. Q Ge, X Huang, J Du, et al. Analysis of drug resistances in the first and second times retreatment pulmonary tuberculosis with smear-positive[J]. Beijing Medical, 2013, 35(12): 989–992.
82. J Yang, D Li, X Zhang, et al. Drug resistance surveillance of Mycobacterium tuberculosis in Shuangliu county[J]. Parasitoses and Infectious Diseases, 2011, 9(4):190–192.
83. S Yan, Y Li, S Xie, et al. Study on the drug resistance of mycobacterium tuberculosis among 5 years in Shunde district [J]. J Prac Med, 2009, 25(4): 639–640.
84. J Yang, H Zhang, X Wang, et al. Distribution characteristics and analysis of genotype drug resistance of drug-resistant gene mutations in Mycobacterium tuberculosis in Deyang district, Sichuan [J]. West China Medical Journal, 2014, 29(8): 1487–1490.
85. J Chen, G Yang, M Luo. Studies on drug resistance and drug resistant spectrum of Mycobacterium tuberculosis in Sichuan area [J]. Modern Prevention Medicine, 2010, 37(17): 3211–3212.
86. Y Yan, C Yang, T Xu, et al. The analysis on drug resistance of mycobacterium tuberculosis for tuberculosis patients in Sichuan province [J]. Journal of Chengdu Medical College, 2012, 7(1): 58–60.
87. B Long, Y Yang, W Wang, et al. Drug resistance of mycobacterium tuberculosis in Sichuan [J]. J Prev Med Inf, 2014, 30(6): 430–433.
88. Y Yang, D Yue, W Gao, et al. Comparison of sputum positive pulmonary tuberculosis in plain and hilly regions of Sichuan[J]. Parasitoses and Infectious Diseases, 2013, 11(4): 204–206.
89. JDeng, QLi, TXiao, et al. Preliminary analysis of the surveillance results on Mycobacterium tuberculosis drug resistance in Zigong city, Sichuan province [J]. Chin Prev Med, 2012, 13(9): 653–656.
90. X Jin, J Hong, H Shen. The present condition of the new registration drug-resistant tuberculosis patients for the last four years in Songjiang district [A]. The committee of Shanghai Preventive Medicine Academic Annual Meeting. 2006: 304.
91. B Sun, Y Hu, F Zhu, et al. Prevalence and analysis of the multidrug-resistant tuberculosis factors in rural areas of North Jiangsu [J]. Chinese Primary Health Care, 2008, 22(11): 66–68.
92. X Yu, X Shen, P Tang, et al. Analysis of multi-drug resistance of Mycobacterium tuberculosis derived from tuberculosis patients in Suzhou city [J]. Journal of Clinical Pulmonary Medicine, 2012, 17(2): 269–271.
93. J Jiang, L X. Analysis on the results of bacteria identification and drug sensitivity test among patients with culture-positive pulmonary tuberculosis in Suzhou [J]. J Tuberc Lung Health, 2014, 3(2): 91–96.
94. X Wang, M Wu, X Shen. Analysis of the drug resistance TB patients in hospital in Suzhou city [J]. Journal of Clinical Pulmonary Medicine, 2009, 14(12): 1674.
95. L Sun, Y Yang, Y Yang, et al. Discussion about the treatment outcome of the 217 cases of initial treatment patients with smear-positive tuberculosis drug resistance in Suihua [J]. Chin J Antitubercul, 2006, 28: 66–67.
96. L Jiang. Analysis of the drug resistance of 256 TB patients in hospital in Taizhou city [J]. Chin J Prev Contr Chron Dis, 2012, 20(5): 589–590.
97. H Wen, M Fan, J Zhang, et al. Actualities of tubercule bacillus drug-resistance in Taiyuan Tuberculosis Hospital[J]. Shanxi Med J, 2006, 5(8): 675–676.
98. F Meng, Q Liu, J Li, et al. Analysis of Mycobacterium tuberculosis drug-resistance situation of sputum culture positive patients visiting Tai’an Tuberculosis Prevention and Treatment Hospital in 2011[J]. Chin J Antituberc, 2012, 34(11): 736–739.
99. P Tai, Y Jiang, Y Wei. Analysis of drug resistance mycobacterium tuberculosis of 159 new smear-positive tuberculosis patients in Taizhou [J]. Med J of Communications, 2012, 26(2): 152–153.
100. B He, S Li, X Zhang, et al. A research on 1297 cases of tuberculosis drug resistance and clinical characteristics [J]. Hainan Medical Journal, 2012, 23(16): 121–123.
101. J Li, A Xu, Z Xu, et al. Discussion about dynamic monitoring of the drug resistance trend in patients with pulmonary tuberculosis [J]. Guide of China Medicine, 2013, 11(16): 117–118.
102. Y Han. Analysis on the drug-resistance of tuberculosis patients in Tangshan city[J]. Hebei Medical Journal, 2014, 36(9): 1406–1408.
103. G Li. Studies on risk factors for drug-resistant tuberculosis and molecular mechanisms of drug-resistant Mycobacterium tuberculosis in Tianjin [D]. Tianjin: Tianjin Medical University, 2010.
104. L Zhang, J Wang, M Wu, et al. Change of drug resistance of first-line anti-TB drugs in Tianjin and clinical supervision mechanism [J]. Chin J Nosocomiol, 2015, 25(2): 336–338.
105. Z Lin, M Hu, S Liu, et al. Initial study of drug resistance of tuberculosis in Tongling area [J]. Journal of Clinical Pulmonary Medicine, 2014, 19(1): 101–103.
106. S Liu, Z Lin, C Li, et al. Identification of Mycobacterium tuberculosis and non-M. tuberculosis and drug-resistant analysis in Tongling area[J]. Journal of Clinical Pulmonary Medicine, 2012, 17(9): 1635–1636.
107. J Shan, W Xue, Y Han. Analysis of 139 cases of smear-positive pulmonary tuberculosis mycobacterial culture and drug susceptibility [J]. Chinese Remedies&Clinics, 2013, 13: 10–11.
108. W Feng. Drug resistance analysis of 337 smear-positive tuberculosis patients [J]. J Med Pest Control, 2014, 30(11): 1285–1287.
109. Q Li, X f Wang, Y f Ding, J L Wang. Analysis of Anti tuberculosis Drugs use and Drug Resistance in our hospital during 2006 ~ 2009[J]. China Pharmacy, 2010, 21(38):3569–3571.
110. LM Wen. Analysis of Drug-resistant tuberculosis epidemic status in Ulanqab city [J]. Modern Women, 2014(12):386–386.
111. H Liu, J L Li, Tuxungu. wulaying Tu, et.al. Distribution analysis of 259 drug resistant tuberculosis cases in Urumchi [J]. Xinjiang Medical Journal. 2013, 43(5):26–28.
112. J Liu, P Hao, L yan Chen, et.al. A survey report of drug susceptibility of M/XDR-TB in Wuxi City [J]. Chinese Journal of Disease Control & Prevention, 2011, 15(11):1001–1003.
113. J H Huang, X F Shen, J Zheng et.al. The drug-resistant characteristics of first line anti-tuberculosis drugs and its related factors among patients with pulmonary tuberculosis registered in Wuxi City [J]. Modern Preventive Medicine, 2015, 42(3): 530–532.
114. P Yun, C W Liu, G Y Zeng. Analysis of 158 resistance trains of Mycobacterium Tuberculosis in Wugang city [J]. Journal of Modern Medicine & Health, 2010, 26(22):3379–3380.
115. J Chen. The Anti-tuberculosis Drugs Susceptibility and Fluoroquinolone Resistance Mechanism in Mycobacterium Tuberculosis in Wuhan [D]. Huazhong University of Science and Technology, 2011.
116. L F Chen, J Z, Jun Chen, et.al. Analysis of drug resistance of 1561 tuberculosis patients in Wuhan [J]. Journal of Clinical Pulmonary Medicine, 2014(09):1638–1641.
117. M L Zhou, Z J Fu, J J Wang, et.al. Analysis on the results of the baseline survey of drug resistance of tuberculosis in Wuhan city [J]. Journal of Public Health and Preventive Medicine, 2012, 23(04):33–38.
118. C Chen, W H Wang, P Peng. Analysis on Factors Influencing Drug Resistance of the First- time retreated Smear -positive Pulmonary Tuberculosis Patients in Wuhan [J]. Medicine and Society, 2013, 26(11):10–12.
119. Y Qi, Z H Shi, Z X Zhang. The Investigation and Analysis of the Current State of Drug Resistance to Tuberculosis in Xi’an Region [J]. Journal of Practical Medical Techniques, 2007, 14(13):1662–1664
120. C G Xiong, Y L Zhang, A Y Yi, et.al. Analysis of Utilization of Anti-Tuberculosis Drugs and Drug Resistance of Mycobacterium Tuberculosis [J]. Evaluation and Analysis of Drug-Use in Hospitals of China, 2013, 13(12):1092–1094.
121. X M Zhang, Z G Liu, L C Zeng, et.al. Preliminary survey on drug-resistance of Mycobacterium tuberculosis in Xi’an city [J]. Disease Surveillance, 2008, 23(10):637–639.
122. G Z B P. Drug Resistance Analysis of 1000 Tibet hospitalized TB Cases [J]. Tibet’s Science and Technology, 2014(2):50–50.
123. J Yang, Yangla, Basangqiongda, et.al. Analysis on the Drug Resistance of Mycobacterium Tuberculosis in Lhasa area, Tibet [J]. Journal of Tibet University, 2014, 29(1):36–39.
124. M C Ge, J H Xu, T Chen, et.al. The Prevalence and Related Factors of MDR-TB in Xian Ning cith[J]. Medical Information, 2014(31).1.
125. M C Ge, J H Xu, T Chen, et.al. Analysis on detection of Global Fund Multi-drug Resistant Tuberculosis project in Xian Ning city [J]. China Hwalth Care & nutrition, 2014(7):4145.
126. J Wang, M Zhu, Y Zhang, et.al. Drug Resistance Analysis of Newly Registered TB Patients [J]. Zhejiang Journal of Preventive Medicine, 2014, 26(12):1240–1242.
127. J L Li, J H Wei, Y C Qi, et.al. Analysis on drug resistance of Mycobacterium tuberculosis during 1998–2008 in Xinjiang [J]. Chinese Journal of Clinicians (Electronic Edition), 2010, 4(7):924–927.
128. Y H Li, J W Liu, X Q Wang, et.al. Analysis of tuberculosis drug resistance in Xinjiang [J]. Bulletin of Disease Control & Prevention (China), 2012(6):17–20.
129. W Jia, W D Wu, X M Gu, et.al. Analysis of Drug Resistance of Tuberculosis in Hotan Area of Xinjiang [J]. Bulletin of Disease Control & Prevention (China), 2008, 23(1):46–48.
130. Y C Qi, J Liu, X M Yu, et.al. Spoligotyping of clinical isolates of Mycobacterium tuberculosis in Xinjiang [J]. Disease Surveillance, 2010, 25(12):951–954.
131. Mireban,Rexiati, X Hu, Y L Xu, et.al. Analysis of drug resistances and strain types in 214 Mycobacterium tuberculosis isolates in Kashi region, Xinjiang [J]. Chinese Journal of Anti tuberculosis, 2012, 34(08):538–541.
132. H L Wu, The drug resistance of Mycobacterium tuberculosis and homology analysis in Xin jiang South area [D]. Xinjiang Medical University, 2014.
133. F L Yang, W J Wang, W Meng, et al. Analysis of the prevalence of drug-resistant TB in Karamay city[J]. Chinese Journal of Anti tuberculosis, 2008, 30(03).
134. W D Wu. The report of WHO Drug Resistance surveillances on tuberculosis in Xinjiang [D]. Xinjiang Medical University, 2010.
135. G M Dai, H R Huang, Qian Liang, et.al. Comparison of molecular epidemiology of Mycobacterium tuberculosis in parts of Xinjiang and Ganshu provinces [J]. Disease Surveillance, 2011, 26(08):592–597.
136. H Li, H J Jin, X G Ma, et.al. Analysis on multidrug-resistant tuberculosis surveillance under DOTS implementation in Xinmi county [J]. Chinese Journal of Anti tuberculosis, 2012, 34(01):1202–1204.
137. Y P Hou,S Z Peng,Y L Peng. Comparison of 130 cases of tuberculosis in patients with drug-resistant cases in Xuzhou urban and rural areas [J]. Journal of Clinical Pulmonary Medicine, 2011, 16(04):627–629.
138. Y G Du, J C Yang, L Zhang, et.al. Analysis of 559 strains mycobacterium tuberculosis resistant to anti-tuberculosis drug in Xuzhou [J]. Chinese Journal of Health Laboratory Technology, 2011(07):1720–1721.
139. Y P Hou, S Z Peng, Y L Peng, et.al. Drug resistance of Mycobacterium tuberculosis in an epidemiological survey in Xuzhou [J]. Clinical Focus, 2011, 26(8):666–668.
140. Y P Wang, Y X Liu, X B Liu, et.al. Drug resistance of Mycobacterium tuberculosis in Yanan [J]. Journal of Shanxi Medical University, 2009, 40(01):58–60.
141. T M Zhang, M Xiong, C Cai, et.al. Clinical analysis of extensive drug resistant tuberculosis [J]. Journal of Clinical Pulmonary Medicine, 2010, 15(08):1130–1131.
142. R B Shao, W Liu, J Yin. Analysis of Mycobacterium bacteria type and drug sensitivity in Yancheng in 2012 [J]. Journal of Nantong University (Medical Sciences), 2014(1):72–74.
143. S Zhang, Y S Chen, W Y Zhou, et.al. Situation analysis of mycobacterium tuberculosis and nontuberculosis mycobacteria drug resistance in Yangzhou [J]. Modern Medicine Journal of China, 2014(4):51–53.
144. C H Liu, YJ Zhou, N Z Tan, et.al. Monitoring and research of drug-resistant TB in Yangjiang [C]. China Anti Tuberculosis Association Science Committee Conference. 2010.
145. X Q Yang, Z L Du. Analysis of drug-resistant Mycobacterium tuberculosis retreatment of 148 Hospital cases [J]. Proceeding of Clinical Medicine, 2008, 17(7):517–518.
146. M T Fang, H M Liu, Y F Su, et.al. Drug-resistant analysis of 628 strains tuberculosis in hospital [J]. Chinese Journal of Anti tuberculosis, 2014, 36(2):131–133.
147. P Li, J M Liu, C Tan. Survey of Mycobacterium tuberculosis drug resistance in Yichang region [J]. Medical Frontier, 2013(15).
148. J Jiang, D B Du, Q Y Xiang. Analysis on hospitalized patients with drug-resistant TB Situation and Trend in Yichang from the year of 2002 to 2006[J]. Chinese Journal of Anti tuberculosis, 2008, 30(04):369–370.
149. J Z Ying, C Y Fang, H Hu, et.al. Analysis on resistance of Mycobacterium tuberculosis in Yongkang city [J]. Zhejiang Journal of Preventive Medicine, 2006, 18(10):24–25.
150. Z M Lu, B C Xin, L J Zhang, et.al. Analysis on hospitalized patients with tuberculosis drug resistance of Mycobacterium tuberculosis in northern of Yu region [J]. China Practical Medicine, 2008, 3(30):213–214.
151. Z Q Yuan, Z X Chen, X L Liu, et.al. Analysis on drug Resistance of Mycobacterium tuberculosis in Yueyang from 2009 to 2010[J]. Practical Preventive Medicine, 2011, 18(09):1777–1778.
152. J Z Hua, L Xu, L Li, et.al. Research on monitoring of drug resistance of Mycobacterium tuberculosis in Yunnan Province [J]. Practical Preventive Medicine, 2006, 13(04):911–912.
153. K H Xu, G Q Chen, Y F Li, et.al. Analysis on drug-resistant tuberculosis in Yuxi, Yunnan Province [J]. Soft Science of Health, 2010, 24(1):68–70.
154. W Liang. Analysis on drug-resistant cases of hospitalized patients with tuberculosis in Zhanjiang [J]. Journal of Qiqihar University of Medicine, 2010, 31(8):1217–1218.
155. Y Shen, W Jiang, R Qin, et.al. Analysis on drug Resistance of patients with tuberculosis in Zhangjiagang city from 2011 to June 2013[J]. The Chinese and foreign health abstract, 2013(32):65–66.
156. T L Zhang, Z K Hong, B H Yan et.al. Baseline survey on drug resistance of smear positive pulmonary tuberculosis case in Zhangzhou city [J]. Strait Journal of Preventive Medicine, 2013, 19(3):12–14.
157. P Lin, J R Pan, J Lin, et.al. Resistance of Mycobacterium tuberculosis isolated from Changle [J]. Chinese Journal of Health Laboratory Technology, 2012(12):3011–3013.
158. Y X Chen, Y J Wu, L Ming, et.al. Observation on secondary resistance of mycobacterium tuberculosis in Changzhi city from 1995 to 2004[J]. Chinese Journal of Anti tuberculosis, 2009, 31(11):677–678.
159. H B Cheng, Y J Wu, X Y Zhang, et.al. Study on primary drug resistance of Mycobacterium tuberculosis in different age patients in Changzhi [J]. Journal of Shanxi Medical University, 2010, 41(12):1060–1062.
160. Z W Liu, H B He, X M Wang, et.al. Analysis on the third TB drug resistance monitoring result in Zhejiang province [J]. Chinese Journal of Preventive Medicine, 2011, 45(2):171–173.
161. Q C Li, L M Wu, M Lu, et.al. Surveillance for tuberculosis drug resistance in Hangzhou, Zhejiang [J]. Disease Surveillance, 2014, 29(03):210–214.
162. D S Xu, S C Zhang, J C Yang, et.al. The analysis on the trend of drug resistance of tuberculosis in Huzhou city, Zhejiang [J]. Chinese Journal of Anti tuberculosis, 2006, 28(02):103–105.
163. Z P Miu, Q Li, H B He, et.al. Developing status and tendency of drug resistant tuberculosis in Zhejiang [J]. Chinese Journal of Anti tuberculosis, 2007, 29(03):215–218.
164. W Wang, X G Hao. Analysis of drug resistance status of mycobacterium tuberculosis in Quzhou from 2011 to 2012[J]. Chinese Journal of Health Laboratory Technology, 2013(18):3601–3602.
165. X Y Yao. Analysis on status and risk factors of multi-drug resistant tuberculosis in Tongxiang[C], Against tuberculosis association academic annual meeting of Zhejiang province.2014.
166. Z Wang, Y H Gong, C D Qian, et.al. Analysis of DNA fingerprints classification and drug resistance of mycobacterium tuberculosis in Zhenjiang [J]. Chinese Journal of Clinical Rational Drug Use, 2014(34):15–17.
167. B Dai, H Jiang, X J Xia, et.al. Sampling survey of drug-resistant tuberculosis in Zhenjiang city [J]. Laboratory Medicine and Clinic, 2013, 10(11):1368–1369.
168. W P Jiang, C H Sun. Analysis of drug susceptibility test results of mycobacterium tuberculosis in Zhenjiang city Chinese Journal of Health Laboratory Technology, 2013(17):3457–3459.
169. L Ding, Y W Sun, et.al. Analysis on the result of drug-resistant of Mycobacterium tuberculosis [J]. Journal of Zhengzhou Railway Vocational & Technical College, 2012, 24(4):43–46.
170. C F Lv, Q Sun, L X Wang, et.al. Case finding of multidrug-resistant tuberculosis through PPM-DOTS in 4 sites in China [J]. Chinese Journal of Anti tuberculosis, 2013, 35(12):955–959.
171. H Lin, J Liu, L Chen, et.al. Drug resistance of tuberculosis from 2003 to 2006 in Chongqing [J]. Journal of Third Military Medical University, 2008, 30(12):1183–1185.
172. X F Yan, P M Cao,H Li, et.al. Data analysis on drug-resistant strains of TB in Chongqing Public Health Center medical from 2011 to 2012[C]. TB branch of the Chinese Medical Association Academic Conference in 2013. 2013.
173. Y Liu, J Liu, K H Jing, et.al. Analysis on monitoring results of resistance of anti-Tuberculosis drug in Chongqing [J]. Modern Preventive Medicine, 2012, 39(03):747–749.
174. Y Liu, Y Ca, W Zhang, et.al. The analysis of current situation on the detection and treatment of MDR-TB patients in six districts and counties of Chongqing city [J]. Chinese Journal of Antituberculosis, 2013, 35(10):808–811.
175. Q Y Wang, D Y Hu, Y Liu, et.al. Study on the status of drug-resistant tuberculosis of urban area of Chongqing [J]. Chongqing Medicine, 2014(22):2913–2915.
176. X L Zhang, L Li, X Wang, et.al. Detection and analysis on 304 strains Mycobacterium tuberculosis in hospital [C]. National tuberculosis conference papers series of Chinese Medical Association in 2008.
177. X L Zhang, L Li, L Zhang, et.al. Analysis of drug resistance of tuberculosis inpatients drug-resistant tuberculosis [J]. Nei Mongol Journal of Traditional Chinese Medicine, 2010, 29(6):68–68.
178. G C Yu, Y Y Chen, Z Y Zhang, et.al. Analysis on drug resistance of pulmonary tuberculosis patients in hospital [J]. Chinese Community Doctors, 2007(7):69–69.
179. Q Wang. Analysis on drug resistance in hospitalized patients with pulmonary tuberculosis [J]. Chinese Journal of Public Health, 2007, 23(06):692–693.
180. K Y Lv, G F Liao, et.al. Analysis on drug resistance and therapeutic effect of pulmonary tuberculosis patients in hospital [C]. Proceedings of the 2006 academic conference of the Chinese Medical Society of tuberculosis. 2006.
181. Y Yang, H B Xie, C H Zheng, et.al. Resistance of Mycobacterium tuberculosis to anti- TB drugs with pulmonary tuberculosis [J]. China Tropical Medicine, 2012, 12(01):64–66.
182. L P Yan, H P Xiao. Clinical monitor and analysis on Ofloxacin resistance of pulmonary tuberculosis [J]. Chinese Journal of Anti tuberculosis, 2009, 31(08):477–480.
183. J F Huang, G F Liao. Analysis of drug resistant of pulmonary tuberculosis inpatients. [J]. China Tropical Medicine, 2011, 11(12):1518–1519.
184. C Wang, Y J Zhang. Analysis of drug resistance of hospitalized tuberculosis case[J]. Jilin Medical Journal, 2009, 30(19):2262–2263.
185. Y X Liu, Z X Mei, L Li, et.al. Analysis of drug resistance on initial treatment and retreatment pulmonary tuberculosis patients in hospital [J]. Jilin Medical Journal, 2011, 32(26):5423–5425.
186. Z Y Wang, R Ji. Clinical Investigation of Patients with Tuberculosis Drug Resistance in Hospital [J]. Medical Information, 2015(15):237.
187. G F Huang, H Gao, N Zhou, et.al. Current situation of drug resistance of tuberculosis patients in special hospitals and discussion on their partition management [J]. Chinese Journal of Health Education, 2013(07):665–666.
188. Z M Liu. Investigation on the situation of multi drug resistant tuberculosis patients in hospital [J]. Health Education on Tuberculosis, 2008(2):28–31.
189. Y Li. Effect of multidrug resistant pulmonary tuberculosis prevention and control project in Zibo City [J]. Journal of Public Health and Preventive Medicine, 2014, 25(02):81–82.
190. Y L Zhao, D Y Zhao, H Li, et.al. Trend of drug resistance to first-line and second-line anti tuberculosis drugs in 7 years of clinical isolates of Mycobacterium tuberculosis [J]. Chinese Journal of Health Laboratory Technology, 2010(02):338–340.
191. J Tang, H H Li, Analysis of initial drug resistance in 49 patients with tuberculosis [J], China Practical Medicine, 2009, 4(6):175–176.
192. Y H Zou, G S Yang, L L Yuan, Analysis of drug resistance and therapeutic effect of 52 cases of recurrent pulmonary tuberculosis [J]. Hainan Medical Journal, 2011, 22(11):77–78.
193. J J Zhou, W F Zeng. Analysis of drug resistance among 86 patients with retreated pulmonary tuberculosis [J]. Journal of Tropical Medicine, 2012, 12(12):1485–1487.
194. Y F Zheng, Z Q Qiu. Analysis of drug sensitivity test results of 97 strains of Mycobacterium tuberculosis in vitro [J]. Strait Pharmaceutical Journal, 2005, 17(6):109–111.
195. W G Liu, C Liu, K L Cui, et.al. Analysis of drug resistance in 104 cases of sputum culture positive pulmonary tuberculosis [J]. Medical information management, 2011, 24(12):321–322.
196. C L Fan, B Wang, B Y Ji, et.al, 105 cases of pulmonary tuberculosis sputum analysis of drug resistance of [J]. Heilongjiang Medicine Journal, 2014(4):850–851.
197. X Y Ma, Y Liu. Analysis of drug resistance and clinical characteristics of 110 cases of tuberculosis destroyed lung [J]. China Foreign Medical Treatment, 2012, 31(35):25–26.
198. D B Du, J Zhong, Drug resistance and treatment of 130 cases of tuberculosis patients treated with tuberculosis control project [J]. Chinese Journal of Clinicians (Electronic Edition), 2010, 04(7):1052–1054.
199. Q Zhou, X Y Shen, X M Fang, et.al, Analysis of drug resistance of 131 strains of Mycobacterium tuberculosis [J]. Zhejiang Journal of Preventive Medicine, 2008, 20(06):23–24.
200. Z L Wang, S L Zheng, D P Zhou, et.al, Drug sensitivity analysis of 134 cases of Mycobacterium tuberculosis [J]. Medical Recapitulate, 2011, 17(09):1434–1435.
201. K E Huang, Q Wang, G Guan, et.al, Analysis of drug resistance and detection of drug resistance genes in 139 strains of Mycobacterium tuberculosis [J]. Zhejiang Clin Med J, 2015, 17(1):117–118.
202. Y Jin. Analysis of drug resistance in 146 cases of primary pulmonary tuberculosis [J]. China Modern Medicine, 2011, 18(2):72–72.
203. X W Yu. Analysis of drug resistance of Mycobacterium tuberculosis in 148 hospitalized patients with pulmonary tuberculosis [J]. China Modern Medicine, 2013(9):484–485. 2011, 18(2):72–72.
204. D F Sun, Y F Chen. Analysis of drug resistance of Mycobacterium tuberculosis in 156 in patients with pulmonary tuberculosis [J]. Chinese Journal of Anti tuberculosis, 2010, 32(08):470–471.
205. Z L Wang, X Liu, Q Yuan. Analysis of drug resistance of 179 cases of Mycobacterium tuberculosis [J]. Journal of Medical Forum, 2011(5):27–29.
206. H Y Jin, S D Ye. Analysis of drug sensitivity test results of 179 strains of Mycobacterium tuberculosis [J]. Chinese Journal of Health Laboratory Technology, 2011(01):152–153.
207. B Gu. Analysis of drug sensitivity test results in 180 patients with pulmonary tuberculosis [J]. Journal of Clinical Pulmonary Medicine, 2007, 12(05):515–516.
208. D J Gao. Analysis of epidemiological characteristics and drug resistance of tuberculosis in Binzhou city during 200 ~ 2011 [D]. Shandong University. 2012.
209. W Yuan, H Zhou, M Zhang, et.al, Analysis of drug sensitivity test results of 200 strains of Mycobacterium tuberculosis [J]. Guizhou Medical Journal, 2006, 30(02):168–169.
210. J Gu, K Wang, S J Tang. Results of drug sensitivity analysis of 201 cases of tuberculosis patients [J]. Journal of Microbes and Infections, 2010, 05(03):146–150.
211. Y Xu. 202 cases of positive sputum culture in patients with tuberculosis of lung resistance [D]. China Medical University, 2009.
212. G X Wu, J J Jiang. Analysis of drug resistance of 209 cases of Mycobacterium tuberculosis [J]. Hainan Medical Journal, 2010, 21(01):109–110.
213. F B Jia, H L Chu. Analysis of the treatment of 210 patients with multi drug resistant pulmonary tuberculosis [J]. Journal of Pathogen Biology, 2013(8):721–723.
214. Z Q Qiu, W Wang, J B Xiu, et.al, 211 tuberculosis patients TB drug resistance status of [J]. Strait Journal of Preventive Medicine, 2010, 16(1):84–85.
215. Y Gao, B Wang, W Zhao, et.al, Analysis of drug sensitivity test results of 220 strains of Mycobacterium tuberculosis [J]. Chinese Community Doctors, 2008(21):224–225.
216. C L Wei, Q Wang, Z G Wang, et.al. Analysis of drug resistance of 223 cases of Mycobacterium tuberculosis [J]. Chinese Journal of Health Laboratory Technology, 2012(09):2222–2224.
217. G S Xing. Analysis of drug sensitivity test results of 224 strains of Mycobacterium tuberculosis [J]. Chinese Journal of Health Laboratory Technology, 2008, 18(08):1597–1598.
218. W F Li. Analysis of drug resistance in 234 cases of sputum positive pulmonary tuberculosis [J]. China Modern Medicine, 2013, 20(36):174–175.
219. Z L Du, Q Wang, F Wang.et.al. Analysis of drug resistance of 253 strains of Mycobacterium tuberculosis [J]. Internal Medicine of China, 2012, 07(4):370–372.
220. H Liu, J L Li, Tuxungu,wulaying. et al. Analysis of drug sensitivity test results of 259 cases of pulmonary tuberculosis in Urumqi city [J]. Occupation and Health, 2014, 30(03):344–345.
221. S Z Wei, Q Y Chen, Q F Liang, et al. Analysis of drug resistance of 262 cases of primary pulmonary tuberculosis patients in different age groups [J]. Chinese Primary Health Care, 2011, 25(10):76–77.
222. W Wang, Z Q Qiu, M Z Lei. Analysis of drug resistance of Mycobacterium tuberculosis in 264 patients with pulmonary tuberculosis after re treatment in outpatient clinic [J]. Strait Journal of Preventive Medicine, 2012, 18(5):85–86.
223. H Fan, G B Liu, L Z Wu, et al. Analysis and Discussion on the screening of 266 patients with suspected multi drug resistant tuberculosis [J]. Journal of Clinical Pulmonary Medicine, 2013, 18(01):101–102.
224. L Jia, H Zhang, C C Wei. Analysis of drug susceptibility test results of 270 cases of Mycobacterium tuberculosis [J]. Frontiers of Medicine, 2013(12):43–44.
225. Z L Tu. Analysis of drug resistance of 272 strains of Mycobacterium tuberculosis [J]. Journal of Public Health and Preventive Medicine, 2007, 18(04):89–89.
226. L Y Chen, L Ma, H H Ru, et al. Analysis of drug resistance of 279 strains of Mycobacterium tuberculosis [J]. Journal of Kunming Medical University, 2014(1):47–50.
227. H B Wang, S H Kang, Y H Liu, et.al. Identification and drug sensitivity test results of 288 strains of Mycobacterium tuberculosis [J]. Chinese cosmetic medicine, 2011, 20(z5):496.
228. Y Xi, L Wang. Analysis of drug resistance of sputum culture positive strains in 301 cases of patients with pulmonary tuberculosis [J]. Chinese medical journal, 2010, 12(11):1561–1563.
229. M S Huang, Z Q Qiu. Analysis of drug sensitivity test results of 306 strains of Mycobacterium tuberculosis [J]. Strait Pharmaceutical Journal, 2007, 19(9):79–80.
230. X Y Zou. Clinical analysis of drug resistance in 318 patients with pulmonary tuberculosis [J]. Internal Medicine of China, 2014, 9(3):310–311.
231. Z Q Huang. Analysis of drug sensitivity test results of 329 strains of Mycobacterium tuberculosis [J]. Hainan Medical Journal, 2006, 17(02):133–133.
232. Y Che, M Yu, G Z Xu, et.al. Analysis of drug resistance to first-line anti tuberculosis drugs in 332 cases of pulmonary tuberculosis patients treated with [J]. Chinese Journal of Health Laboratory Technology, 2014(23):3481–3483.
233. C H Gu, Q Wang, F R Wang, et.al. Analysis of drug resistance of first-line anti tuberculosis drugs in 340 cases of tuberculosis [J]. Journal of Ningxia Medical University, 2013, 35(01):74–76.
234. Z P Zhang, X X Li, Y F Wen, et.al. Analysis of drug resistance characteristics of 342 sputum culture positive pulmonary tuberculosis strains [J]. Modern Preventive Medicine, 2015, 42(03):522–525.
235. X H Luo, X L Yang. Analysis of drug sensitivity of 352 strains of Mycobacterium tuberculosis [J]. Journal of Clinical Pulmonary Medicine, 2011, 16(05):723–724.
236. X Liang, X L Yu, X Y Zheng. Analysis of drug resistance of 364 strains of Mycobacterium tuberculosis [C]. Science and technology innovation and industrial development. 2010.
237. Mireban,rexiati. Analysis of drug resistance in 371 cases of pulmonary tuberculosis [D]. xinjiang medical university, 2012.
238. D P Zhou, H J Li, L Sha. Clinical analysis of drug resistance of 378 strains of Mycobacterium tuberculosis [J]. Chinese Journal of practical medicine, 2011, 38(22):111–112.
239. Q H Wu, X L Wang. Analysis of drug resistance of Mycobacterium tuberculosis in 388 Cases [J]. Journal of Clinical Pulmonary Medicine, 2011, 16(04):550–551.
240. L Zhang. 395 cases of positive sputum culture analysis of pulmonary tuberculosis patients hospitalized for Drug resistance situation [D]. Jilin University, 2014.
241. H Y Yin, Y D Liu, H P Xiao, et.al. Correlation analysis of drug sensitivity and clinical features in 417 cases of tuberculosis patients [J]., Chinese Journal of Practical Internal Medicine, 2009(12):1100–1102.
242. Y W Zou, T Wang, L Yang, et.al. 431 cases of retreatment patients of Mycobacterium tuberculosis on line analysis of anti-tuberculosis drug resistance [J]. Journal of Clinical Pulmonary Medicine, 2014(09):1729–1730.
243. M Y Qu, W J Feng, Y K Liu. 471 cases of tuberculosis patients tuberculosis drug susceptibility analysis and prevention of [J]. Medical clinical research, 2013, 30(9):1800–1802.
244. J X Liu, D D Liu. Analysis of drug resistance of 493 cases of Mycobacterium tuberculosis [J]. Journal of tuberculosis and lung health, 2013, 30(9):1800–1802.
245. Z Y Zhang, S H Tu, R Y Tu, et.al. Analysis of drug sensitivity test results of 509 strains of Mycobacterium tuberculosis isolates [J]. Laboratory medicine and laboratory medicine, 2006(S1):607–608.
246. Y D Liu, S J Tang, L J Jing, et.al. Analysis of drug resistance of 518 strains of Mycobacterium tuberculosis in the first and second line drugs [C]. Proceedings of the 2011 academic conference of the tuberculosis branch of the Chinese Medical Association. 2011.
247. J F Wu, R B Shao, W Liu, et.al. Analysis of drug sensitivity test results of 534 strains of Mycobacterium tuberculosis [J]. Modern Preventive Medicine, 2014, 41(07):1334–1336
248. C H Cai, G Y Zeng, L P Li, et.al. Analysis of drug resistance of 540 strains of Mycobacterium tuberculosis [J]. Zhejiang Clin Med J, 2013(7):1075–1076.
249. Y H Tan, S L Yi, Z Liang. Analysis of drug resistance of 591 strains of Mycobacterium tuberculosis [J]. Chinese Journal of Anti tuberculosis, 2009, 31(03):157–159.
250. D F Xu. Analysis of drug resistance of 599 strains of Mycobacterium tuberculosis [C]. Anhui Medical and Pharmaceutical Journal, 2009:117–119.
251. X L Zhang, Q Wu, L Zhang, et.al. A retrospective review of drug resistance trends of 609 cases of tuberculosis patients in the hospital with the drug resistance of Mycobacterium tuberculosis [J]. Jilin Medical Journal, 2010, 31(4):510–511.
252. L Q An, J X Wang, Z Sun, et.al. Analysis of drug resistance of 656 strains of Mycobacterium tuberculosis [J]. Chinese Journal of Prevention and Control of Chronic Diseases, 2011, 19(01):36–37.
253. W P Jiang, C H Sun. Analysis of drug resistance of 680 strains of Mycobacterium tuberculosis [J]. Journal of Public Health and Preventive Medicine, 2013, 24(04):102.
254. C F Yang. Analysis of drug resistance of 702 strains of Mycobacterium tuberculosis [J]. China Practical Medicine, 2008, 3(23):80–81.
255. D P Fan, G Chen, S H Wu, et.al. Analysis of drug resistance of Mycobacterium tuberculosis in 723 patients with tuberculosis [J]. Zhejiang Journal of Preventive Medicine, 2013, 25(12):42–44.
256. W Guo, Q H Zhang, Q N Cheng, et.al. The positive analysis of drug resistance in patients with pulmonary tuberculosis and 728 cases of sputum culture [J]. Anhui Medical Journal, 2011, 32(07):983–984.
257. L N Feng, Y D Han, Z X Yu. Drug sensitivity test of Mycobacterium tuberculosis in 750 cases of tuberculosis [J]. Chinese Journal of Gerontology, 2013, 33(15):3759–3760.
258. R R Zheng, X D Zhang, C J Huang. Analysis of drug resistance of 875 strains of Mycobacterium tuberculosis [J]. Modern Preventive Medicine, 2008, 35(23):4697–4698.
259. Y Luo, Q E Xie. Analysis of drug resistance of 921 strains of Mycobacterium tuberculosis [J]. Journal of Gannan Medical University, 2012, 32(1):76–77.
260. H B He, Z W Liu. Analysis of drug resistance in 938 cases of Mycobacterium [C]. Data compilation of the Symposium on the diagnosis and treatment of multi drug resistant tuberculosis. 2005.
261. Z W Liu, H B He, Z P Miu, et.al. Analysis of drug resistance of 984 strains of Mycobacterium tuberculosis [J]. The Journal of the Chinese Anti tuberculosis Association, 2007, 29(02):167–170.
262. W Qin, W Z Ou, K W Luo, et.al. Analysis of 996 cases of tuberculosis patients in resistance to rifampin and isoniazid [J]. Guizhou Medical Journal, 2014(01):66–68.
263. L W Ma. Analysis of drug resistance of 1008 strains of Mycobacterium tuberculosis [J]. China Practical Medicine, 2007, 2(35):128–129.
264. X Y Wang. Study on drug resistance of 1011 strains of Mycobacterium tuberculosis [J]. Laboratory Medicine and Clinic, 2010, 7(08):712–713.
265. S L Li, W S Chen, Y P Huang, et.al. Analysis of drug resistance of 1040 strains of Mycobacterium tuberculosis [J]. China Tropical Medicine, 2014, 14(06):749–750.
266. W M Chen, X Chen, Z G Wang, et.al. Analysis of drug resistance in 1056 cases of pulmonary tuberculosis [J]. Journal of Clinical Pulmonary Medicine, 2012, 17(02):296–297.
267. W M Li, L S Tang, Y Li, et.al. Analysis of drug resistance in 1067 cases of tuberculosis [J]. Guangxi Medical Journal, 2014(06):780–782.
268. J Yao, Y H Li, J R An. Analysis of drug resistance of 1083 strains of Mycobacterium tuberculosis [J]. Disease prevention and control bulletin, 2011(3):52–52.
269. M L Gong, J B Li. Analysis of drug resistance of 1191 strains of Mycobacterium tuberculosis [J]. Journal of Diseases Monitor & Control, 2013, 7(1):49–49.
270. Y G Xiang, W Z Deng, X F Peng, et.al. Analysis on the results of single and combined drug sensitivity test of 1684 strains of Mycobacterium tuberculosis [J]. Practical Preventive Medicine, 2007, 14(02):536–537.
271. H Ding, Y L Chen. Analysis of drug resistance of 1800 strains of Mycobacterium tuberculosis to 10 kinds of anti-tuberculosis drugs [J]. Acta Universitatis Medicinalis Nanjing(Natural Science), 2008(10):1361–1364.
272. W Q Wu, J H Zhan, Q L Liu, et.al. Analysis of sputum culture and drug sensitivity test of 1976 cases of pulmonary tuberculosis patients [J]. Modern Preventive Medicine, 2013, 40(19):3687–3690.
273. Y M Cheng, X R Zeng, Y H Wu. Analysis of TB drug resistance of culture positive pulmonary tuberculosis patients in Quanzhou city during 1991–2005 [J]. Strait Journal of Preventive Medicine, 2010, 16(2):76–78.
274. S W Wang, X Z. Analysis of drug resistance of tuberculosis in Nanjing area from 1996 to 2006 [J]. The Journal of the Chinese Antituberculosis Association, 2008, 30(05):406–408.
275. H Y Yin, Y R Zhang, H P Xiao, et.al. Analysis of four-line drug resistance of tuberculosis in hospital during 2003–2006 [C]. Proceedings of the 2008 National Symposium on tuberculosis. 2008.
276. H Y Wang, Y Wang, C B Yu, et.al. Detection and analysis of drug resistance of Mycobacterium tuberculosis in Shandong province from 2004 to 2007 [J]. Preventive Medicine Tribune, 2008, 14(12):1075–1076.
277. C Y Liao, Z G Huang, T G Chen, et.al. The trend of drug resistance of Mycobacterium tuberculosis clinical isolates from 2004 to 2009 [J]. Journal of Clinical Pulmonary Medicine, 2011, 16(08):1212–1213.
278. W Wang, W J Zhou, Z Y Dou, et.al. Analysis of drug resistance of Mycobacterium tuberculosis in Qingdao city from 2004 to 2007 [J]. Journal of North China Coal Medical University, 2009, 11(5):642–643.
279. Liu, G J Gao, H F Chen, et.al. Analysis of drug resistance of Mycobacterium tuberculosis in hospitalized patients with pulmonary tuberculosis from 2006 to 2004 [J]. Hebei Medical Journal, 2007, 29(11):1260–1261.
280. G C Yu, X X Zhang, Y H Meng. Investigation and analysis of initial drug resistance in hospitalized patients with pulmonary tuberculosis from 2006 to 2005 [J]. Jilin Medical Journal, 2008, 29(1):52–53.
281. J Liu, G H Jing, Y Liu, et.al. Analysis of tuberculosis drug resistance surveillance in Chongqing city in 2005 [J]. Laboratory Medicine and Clinic, 2009, 6(12):963–965.
282. D L Liu, S Y Liu, W L Zhang, et.al. Surveillance of drug resistance of tuberculosis in Huairou District of Beijing city from 2006 to 2008 [J]. Capital Journal of Public Health, 2010, 4(1):22–24.
283. G J Gao, S C Wu, H Q Di, et.al. Investigation on drug resistance of pulmonary tuberculosis in hospital from 2006 to 2009 [J]. Journal of Clinical Pulmonary Medicine, 2011, 16(11):1797–1797.
284. J P Wang, Y J Liu, D Wu. Investigation and analysis of drug resistance in the primary treatment of pulmonary tuberculosis in a hospital from 2007 to 2008 [J]. Chongqing Medicine, 2010, 39(21):2944–2945.
285. D Y Zhao, Y L Zhao, H Li, et.al. Analysis on drug resistance of Mycobacterium tuberculosis in Henan area from 2007 to [J]. Chinese Journal of Health Laboratory Technology, 2010, 20(05):1109–1111.
286. Y Q Zhu, C H Sun. Investigation on drug resistance of pulmonary tuberculosis patients in Danyang city during 2008–2009[J]. Chinese Journal of Ethno medicine and Ethnopharmacy, 2010, 19(15):116–116.
287. L Y Wang. Analysis of drug resistance surveillance of tuberculosis in Taizhou city during 2008–2009 [C]. China Anti Tuberculosis Association Science Committee Conference. 2010.
288. D J Gao. Analysis of epidemic characteristics and drug resistance of tuberculosis in Binzhou city from 2008 to 2011 [D]. Shandong University. 2012.
289. Q Li, Y B Zhang, M Lei. Changes of drug resistance of Mycobacterium tuberculosis during 2008–2011 [J]. Chinese Journal of Nosocomiology, 2013, 23(19):167–167.
290. X Y Chen. Analysis of baseline survey of drug resistance of Mycobacterium tuberculosis in Wuxi city in 2008 [J]. Chinese and foreign health Abstracts. 2010, 07(29):147–148.
291. P Luo, T Zhang, Z Gao. The study of drug resistance of retreatment tuberculosis patients in Beijing 2009–2010[J]. Chinese Journal of Antituberculosis,2012,34(11):704–707
292. Y Zhou, Y Man. Trend and retrospective study of tuberculosis mycobacterium for the first-line anti-TB drugs in Nanyang from 2009 to 2011 [J]. Medical Frontier, 2012, 02(8):344–345.
293. D Fan, Y Zhang, Q Xia. Analysis of 636 cases of drug-resistant tuberculosis in Hangzhou city from 2010 to 2011[J]. Chinese Journal of Anti tuberculosis, 2012, 34(11):750–751.
294. F Meng. Analysis on drug-resistance of Mycobacterium tuberculosis among some patients with positive sputum culture in Tai’an City during 2010–2011 [J]. Preventive Medicine Tribune, 2014, 20(2):84–87.
295. X Hao, W Wang, and W X. Drug resistance of Mycobacterium tuberculosis in smear positive pulmonary tuberculosis patients in Quzhou, Zhejiang, 2010–2012 [J]. Disease Surveillance, 2013, 28(6):467–469.
296. J Shen, J Liu, L Chen. Analysis of drug-resistant TB in three gorges reservoir from 2010 to 2013[J]. Chinese Journal of Antituberculosis, 2014, 36(7):599–602.
297. Y Lei, X Chen, S Mei. Drug sensitivity test of mycobacterium tuberculosis in mountainous area of Lishui during 2010–2012[J]. Chinese Journal of Health Laboratory Technology, 2014, 24(1):133–137.
298. J Zhang, T Wu, J Shi. Analysis of results of drug resistance monitoring among smear positive pulmonary tuberculosis patients in Donghai County from 2011 to 2012[J]. Chinese Journal of School Doctor, 2014, 28(3):191–192.
299. M Yang, L Zhao, Z Gao. Analysis on Drug Resistance of Newly Registered Cases of Tuberculosis in Two Surveillance Counties, Baoshan City, 2011[J]. Preventive Medicine Tribune, 2013, 19(2):128–130.
300. Y Man, Y Zhou. Analysis of TB drug resistance surveillance results in Nanyang [J]. Chinese Journal of Health Laboratory Technology, 2012, 22(7):1704–1707.
301. H Ning, S Tian, B Ning. Analysis of TB drug resistance surveillance of 138 sputum culture positive pulmonary tuberculosis patients in Xunchang city during 2012–2013[J]. Preventive Medicine Tribune, 2014, 20(1):58–59.
302. L Zheng, Q Zhang. Analysis of TB drug resistance surveillance results of tuberculosis (TB) first-line anti-TB drugs in Zhongshan city in 2012 [J]. Jilin Medical Journal, 2013, 34(20):4049–4050.
303. Y Li, Z Pa, N Liu. The drugs resistance status of pilmonary tuberculosis patients in Bortala Mongolia Automomous Prefecture in 2013[J]. Chinese Journal of Antituberculosis, 2015, 34(7):389–389–392.
304. H Fan, G Liu, L Wu. Analysis and investigation of 266 suspicious tuberculosis patients with multidrug resistance [J]. Journal of Clinical Pulmonary Medicine, 2013, 18(1):101–102.
305. B Lin, L Chen, X Zhang. Analysis on drug resistance of 2016 cases of mycobacterium tuberculosis [J]. China Health Care and Nutrition, 11(20):4711–4711.
306. K Li. Analysis of TB drug resistance surveillance of 2059 cases of mycobacterium tuberculosis [J]. Journal of Public Health and Preventive Medicine, 2007, 18(02):73–74.
307. X Zhang, Yang H. Analysis of 2144 drug resistant Mycobacterium tuberculosis strains [J]. China Tropical Medicine, 2014, 14(12):1480–1482.
308. X Cheng, Y Li, Y Rao. Drug resistance situation in 2271 tuberculous patients and epidemiological characteristics analysis [J]. Chongqing Medicine, 2015, 44(12):1635–1637.
309. T Zhang, Y Wang, K Luo. An Analysis on Resistance Status of 2327 Strains of Bacillus tuberculosis to Multi-drugs [J]. Journal of Guiyang Medical College, 2009, 34(6):643–645.
310. Chen J, Chen Z, Zhang T. Analysis on drug resistance of 2433 cases of mycobacterium tuberculosis [J]. Guizhou Medical Journal, 2013, 37(03):259–260.
311. M Chen, L Zhang, Y Li. Analysis of drug-resistance situation on 2672 pulmonary tuberculosis inpatients [J]. Jiangsu Journal of Preventive Medicine, 2014, 25(2):36–38.
312. C Liu, Y Liu. Analysis of drug-resistance on 3653 sample of tuberculosis patients [J]. Modern Preventive Medicine, 2011, 38(04):725–727.
313. L Zhang, M Tao, F Du. Analysis of hospitalized cases of 4567 cases of mycobacterium tuberculosis [J]. Anhui Journal of Preventive Medicine, 2013, 19(6):457–458.
314. L Dang, L Wei, R Fang. Analysis of the drug-resistant status and risk factors of 4721 cases of hospitalized tuberculosis patients [J]. Chinese Journal of Anti tuberculosis, 2014, 36(1):49–54.
315. X Shi, L Jie, Q Wang. Analysis of sputum culture tuberculosis bacterium results of 7512 cases [J]. Xinjiang Medical Journal, 2009, 39(8):45–47.
316. W Jia, W Wu, W Zhang. The report of WHO drug resistance surveillance on tuberculosis, Xinjiang [J]. Journal of the Chinese Anti tuberculosis Association, 2008,30(4):307–310.
317. J Liu, H Pei, L Chen. Analysis of Drug susceptibility result of M/XDR-TB in Wuxi city [J]. Chinese Journal of Disease Control & Prevention, 2011, 15(11):1001–1003.
318. X Yu, Y Gu, Y Luo. Analysis of drug resistance of 101 strains Mycobacterium tuberculosis in Aba prefecture people’s hospital [J]. The Chinese and foreign health abstract, 2011, 08(12):95–96.
319. D Wang, X Pan, H Chai. Drug resistance to first-line anti-tuberculosis drugs of Mycobacterium tuberculosis in Anhui province [J]. Anhui Medical Journal, 2012, 33(9):1131–1134.
320. Xu D, Wang Q, Li N. Resistance analysis of the first and second line anti-TB drugs in 420 clinical Mycobacteria tuberculosis isolates from Anhui Province [J]. Chinese Journal of Zoonoses, 2014, 30(1):54–57.
321. G Liu. Analysis of Drug Resistance of Pulmonary Tuberculosis Patients in Anhui Pulmonary Hospital from 2003 to 2004[J]. Anhui Journal of Preventive Medicine,2005,11(1):7–8.
322. X Kan, K Wang, J Yang. The survey on the status of drug resistance in Mycobacterium tuberculosis in Anhui province [J]. Anhui Medical Journal, 2009, 30(5):507–509.
323. D Xu, Q Wang. Monitoring of mycobacterium tuberculosis for the first and second-line anti-TB in Anhui Specialized subject hospital[C]. Scientific research and TB peak BBS.2014.
324. Z Guo, Z Wang, X Liu. The preliminary analysis and counter measures of the drug resistance of Mycobacterium tuberculosis in Bengbu [J]. Journal of Bengbu Medical College2013, 38(4):469–470.
325. J Li, Z Lv, H Duan. Analysis of Drug Resistance of Pulmonary Tuberculosis in Baotou city in 2008, 2009 [J]. The Chinese and foreign health abstract, 2010, 07(14):276–277.
326. B Tao. Survey on the situation of drug-resistance of Mycobacterium tuberculosis in Beihai, Guangxi [J]. Journal of Clinical & Experimental Medicine, 2012, 11(12):966–968.
327. X Jiang. Study on the genotyping and drug-resistance epidemiology of Mycobacterium tuberculosis in Beijing [D]. Beijing Chest Hospital.2012
328. Z Xie, H Gao, G Wu. Analysis of TB drug resistance situation in Beijing Specialized subject hospital in 2008–2010 [J]. Medical Information, 2011, 24(17):5702–5704.
329. Y An, B Ding, J Zhu. Report of WHO survey on drug-resistant tuberculosis in Beijing [J]. Journal of the Chinese Antituberculosis Association, 2007, 29(6):475–478.
330. Z Zhang, Y Cui, T Gao. Analysis of initial drug resistance of Tuberculosis in Beijingchangping district during 2004–2005[J]. Chinese Journal of Anti tuberculosis, 2007, 29(01):96–97.
331. H Zhang, A Zhang, P Zhao. Analysis of the status and risk factors of drug resistant tuberculosis in Chaoyang district in Beijing [J]. Chinese Journal of Anti tuberculosis, 2009, 31(4):218–222.
332. P Luo, T Zhang, Z Gao. Study of retreatment TB drug resistance situation in Beijing[C]. Professional committee of the tuberculosis control conference, 2012.
333. J Chen, T Zhang, C Cai. Drug resistance on different types of relapsing pulmonary tuberculosis [J]. Modern Preventive Medicine, 2014, 41(7):1339–1341.
334. J Pu, W Gao, L Xie. Analysis on primary drug resistant rate in hospitalized patients with pulmonary tuberculosis in different periods [J]. Chinese Journal of Antituberculosis, 2009, 31(9):523–525.
335. B Lin, L Chen, X Zhang. Analysis on TB drug resistance situation of tuberculosis patients with different professional and culture [J]. China Health Care Nutrition, 2013, 23(3):893–895.
336. Z Zhang, K Qin, Y Wu. Clinical Analysis of Species Identification and Drug Susceptibility Tests of Acid-Fast Bacilli in Chang Zhou Region[C]. Proceedings of the 12th week of pharmacists in jiangsu province, 2012.
337. D Yang, Y Zhang, A Luo. Analysis on TB drug resistance situation of 826 tuberculosis cases in Chengdu city[C]. Defense disease association in national academic conference of the 80th anniversary. 2013
338. G Wu, X Zhou, H Luo. Analysis of the Anti-tuberculosis drugs resistance of Mycobacterium tuberculosis in Chengdu area [J]. Modern Preventive Medicine, 2010, 37(9):1753–1754.
339. L Chen, Q Zhao, T Luo. Analysis on drug resistance situation of 688 strains of mycobacterium tuberculosis in Chengdu city [J]. Chinese Journal of Public Health, 2011, 27(11):1495–1496.
340. Luo A, Ye F, Luo H. Analysis of Drug Resistance Treating Mycobacterium Tuberculosis in Chengdu [J]. West China Medical Journal, 2010, 25(10):1854–1856.
341. J Ai, Y Dan. Analysis of drug susceptibility of smear-positive TB in Chibi city in 2011[J]. China’s rural health, 2012(Z2):482–482.
342. J Wang, Q Wang, Y Du. Analysis of Drug Resistance of 160 strains initial and retreatment of tuberculosis [J]. Chinses Journal of Clinicians, 2012, 6(3):161–163.
343. C Gao, R Zhang, X Xi. Early retreatment smear-positive pulmonary tuberculosis patients and extensively drug-resistant MDR Survey [J]. Frontiers of Medicine, 2014, 34(11):239–240.
344. A Du. Bacteria identification and drug resistance analysis [J]. China Practical Medicine, 2015, 10(15):194–195.
345. Y Liu, R Liu, Z Li. Analysis of initial Drug Resistance of initial smear-positive pulmonary tuberculosis patients [J]. Jilin Medical Journal, 2012, 33(18):3900–3900.
346. Z Shi, R Li, B Duan. Monitoring result analysis on resistance of 87 cases of tubercle bacillus in Dali [J]. Chinese Journal of Pest Control, 2012, 28(12):1315–1317.
347. Q Song, C Cai, Y Ren. Risk factors for MDR- TB and XDR- TB in Dalian patients [J]. Journal of Dalian Medical University, 2015, 37(1):45–48.
348. X Yu, B Xin. Characteristic analysis of TB drug resistance in Daqing area [J]. Contemporary Medicine, 2013, 19(13):161–163.
349. J Liu, L Cui, F Wang. Analysis of TB drug resistance of smear-positive pulmonary tuberculosis patients [J]. Contemporary Medicine, 2011, 17(6):162–163.
350. J Zhu, W Wang, X Wang. Epidemic Pattern of Drug-Resistant Tuberculosis and its Risk Factors in Deqing County [J]. Zhejiang Journal of Preventive Medicine, 2009, 21(9):6–8.
351. H Rong. Drug sensitive experiment and clinical analysis of drug-resistant mycobacterium tuberculosis [J]. Jilin Medical Journal, 2009, 30(6):501–501.
352. Y Lei, A Liu, X Hou. Drug-resistance distribution on tuberculosis strains and research of correlation between multidrug-resistance phenotypes of mycobacterium tuberculosis and Beijing genotypes strains [J]. Modern Preventive Medicine, 2013, 40(3):528–530.
353. W Lin, G Li. Strategy and analysis of drug resistance situation of pulmonary tuberculosis patients[C]. National academic conference drug-resistant TB in 2009, 2009.
354. X Chen, M Zheng, L Zhong. Analysis on Drug Resistance of 300 Cases Pulmonary Tuberculosis [J], Hebei Medicine. 2010,16(2):172–174.
355. C Niu, J Ma, Z Jia. Analysis on Drug Resistance of 342 Cases Pulmonary Tuberculosis [J]. Clinical Focus, 2012, 27(1):71–74.
356. N Li, L Chen, H Zhang. Drug analysis on mycobacterium tuberculosis of pulmonary tuberculosis patients [J]. Chinese Journal of Clinical Laboratory Science, 2010, 28(05):387–388.
357. Yuan W, Yang J, Lei S. Analysis of monitoring results of multi-drug resistant pulmonary tuberculosis patients [J]. Laboratory Medicine & Clinic, 2013, 10(13):1635–1637.
358. H Li. Drug analysis pulmonary tuberculosis patients [J]. Chinese Journal of Primary Medicine and Pharmacy, 2011, 18 (22):3106–3107.
359. Z Guan. Tuberculosis patients with drug-resistant and impact factors [J]. Chinese Journal of Medicinal Guide, 2013, 15(7):1236–1237.
360. H Duan. Drug resistance in tuberculosis sputum: a report of 568 cases [J]. Journal of Shanxi Medical University, 2010, 41(7):634–636.
361. H Wang, X Liu, L Liu. Analysis of 139 cases of drug resistance of mycobacterium [J]. Journal of Huaihai Medicine, 2013, 31(5):397–399.
362. W Dan, Y Xiao, K Luo. Analysis of infectious status of mycobacterium and drug resistance of mycobacterium tuberculosis [J]. Journal of Clinical Pulmonary Medicine, 2014, 19(6):1046–1049.
363. L Yuan, J Li, Y Zou. Analysis on drug-resistance of smear-positive tuberculosis patients in Foshan city [J]. Journal of Tuberculosis and Lung Health, 2014, 3(1):40–46.
364. S Wei, Y Song, Chen Q. Survey of 160 drug resistant pulmonary tuberculosis patients in Longyan City, Fujian [J]. China Tropical Medicine, 2011, 11(9):1094–1096.
365. Q Chen, J Zheng, Q Liang. Study on tuberculosis drug resistant surveillance in nine sites in Fujian province[C]. National conference of defense tuberculosis in 2011, 2011.
366. Q Chen, S Lin, Q Liang. Tuberculosis drug resistance surveillance report in Fujian province [J]. Chinese Journal of Antituberculosis, 2013, 35(7):511–515.
367. Y Cheng, X Ruan, X Zeng. The analysis of tuberculosis epidemic situation and the effect of control program in Quanzhou, Fujian [J]. Chinese Journal of Antituberculosis, 2013, 35(8):609–615.
368. Y Zhao, Q Chen, Q Liang. Drug resistance surveillance analysis of 451 cases in Fujian southwest province tuberculosis[C]. National conference of defense tuberculosis in 2011, 2011.
369. Y Zhu, L Zhang, L Chen. Drug resistance analysis of tuberculosis in Fuzhou city during 2006–2010 [J]. Strait Journal of Preventive Medicine, 2013, 19(4):30–31.
370. Z Cao, Q Li. Analysis on initial drug-resistance of initial tuberculosis patients [J]. The Chinese and foreign health abstract, 2011, 08(21):232–234.
371. J Ma, Y Zhang, G Tan. Drug and therapeutic effect analysis of retreatment tuberculosis patients [J]. Chinese Journal of Lung Diseases, 2015, 8(02):61–63.
372. L Zhao. Drug analysis and control measures of retreatment tuberculosis patients[J]. Journal of Clinical Pulmonary Medicine, 2013, 18(04):753–754.
373. M Yang, C Li, X Xiang. Analyzing of Treatment Effect in Recurrence of Pulmonary Tuberculosis for drug-Resistant [J]. Journal of Modern Clinical Medicine, 2013, 39(5):341–342.
374. Y Chen, X Guo, W Chen. Analysis of drug resistance pattern in retreatment patients with pulmonary tuberculosis [J]. International Journal of Laboratory Medicine, 2012, 33(7):812–813.
375. Liang B, Wei Y, Wu H. analysis on drug characteristic and relevant factor of retreatment smear-positive patients with pulmonary tuberculosis [J].
376. Y Luo, J Yang. A study on characteristics of rifampin-and isoniazid-resistance genes of Mycobacterium tuberculosis in Ganzhou region [J]. International Journal of Laboratory Medicine, 2014, 35(9):1136–1137.
377. B Liu. Analysis on drug situation and reason of retreatment patients with pulmonary tuberculosis in Ganzhou city [J]. Chinese Journal of Antituberculosis, 2006, 28(S1):24–24.
378. J Peng, L Yi, C Hu. Analysis on current situation of tuberculosis control project and initial drug resistance rate of pulmonary tuberculosis in Dongguan city [J]. Bulletin of Chinese Antituberculosis Association, 2005, 20(1):330–332.
379. R Zhang, Q Zhong, Chen S. Analysis on drug resistance situation of 348 cases pulmonary tuberculosis in Foshan city, Guangzhou [J]. Chinese Journal of Antituberculosis, 2011, 33(08):516–517.
380. Q Zhong, J Yin, M Qian. Baseline investigation of tuberculosis drug-resistant of Guangdong province [J]. Chinese Journal of Antituberculosis, 2011,33(7):393–402.
381. C Liu, Y Zhou. N Tan. Drug resistance surveillance analysis of tuberculosis in Yangjiang city, Guangzhou [J]. Chinese Journal of Antituberculosis, 2010, 32(09):606–608.
382. W Huang. Development of TB drug resistance in Zhaoqing area of Guangdong Province [J]. Journal of Clinical Pulmonary Medicine, 2005, 10(3):293–294.
383. H Liang, J Liang. Analysis on clinical drug resistance of pulmonary tuberculosis in Shunde District, Guangzhou [J]. Chinese Journal of Ethnomedicine and Ethnopharmacy.2014 (12):82–83.
384. H Wei, X Long, J Ling. The characteristics of the genes mutations in rifampin and isoniazid resistant Mycobacterium tuberculosis clinical isolates from Baise district, Guangxi autonomous region [J]. The Journal of Practical Medicine, 2015, 31(05):731–734.
385. S Huang, S Lan, F Liu. Survey on drug resistant pulmonary tuberculosis and analysis of risk factors to drug resistance of retreatment cases in Guangxi [J]. Chinese Journal of Anti tuberculosis, 2013, 159(5):993–1003.
386. Z Zheng, J Wei, B Wei. Survey of drug resistance situation of smear-positive pulmonary tuberculosis patients [J]. Chinese Journal of Anti tuberculosis, 2012, 34(06):404–406.
387. W Feng. Treatment outcomes by drug resistance and HIV status among tuberculosis patients in Ho Chi Minh City, Vietnam [J]. China Tropical Medicine, 2014, 14(9):1129–1130.
388. T Li, Z Fang, W Ke. Analysis of tuberculosis patient’s sputum examination and drug sensitive test in Hefei [J]. Chinese Journal of Disease Control & Prevention, 2010, 14(12):1246–1248.
389. H Zhang, L Wang, Y Bao. Analysis of screening data of suspicious multidrug resistant tuberculosis cases in Hefei [J]. Anhui Journal of Preventive Medicine, 2014, 20(6):437–439.
390. J Xu, K DeRiemer, Li H. prevalence and risk factors of drug resistant tuberculosis in 5 counties(cities) in Henan of 2007 [J]. Modern Preventive Medicine, 2012, 39(2):273–278.
391. H Li, X Ma, J Shi. Analysis on bacteriology examination results of epidemiological sampling survey for tuberculosis sites of Henan province in 2010[J]. Chinese Journal of Antituberculosis, 2013, 35(1):22–26.
392. B Ma, J Sun, H Liu. Analysis of drug resistance of 1119 Mycobacterium tuberculosis strains in Puyang [J]. Chinese Journal of Anti tuberculosis, 2014, 36(4):279–285.
393. D Zhao, H Yang, X Ma. Survey on etiology of tuberculosis and drug-resistance of Mycobacterium tuberculosis in Weishi county, Henan province [J]. Disease Surveillance, 2011,26(6):446–449.
394. Y Zhao, X Ma, H Li. Genotyping characteristic and drug resistance of Mycobacterium tuberculosis isolated in Weishi county of Henan province[J]. Journal of Zhengzhou University, 47(5):691–693.
395. Y Xie, F Li, X Lv. WHO TB drug resistant survey in Heilongjiang province [J]. Journal of the Chinese Anti tuberculosis Association, 2008,30(5):395–398.
396. H Yang, Y Zou, Y Shi. A comparison study on the multi-drug resistance of tuberculosis in Huzhou city [J]. Zhejiang Journal of Preventive Medicine, 2014, 26(3):233–236.
397. H Li. Analysis on drug resistance situation of pulmonary tuberculosis in Hecheng district, Huang city [J]. China Health Industry, 2014(20):156–156.
398. S Xie, J Zhu, R Wang. Analysis of drug resistance of pulmonary tuberculosis patients in HuaiBei from 2009 to 2010[J]. Journal of Clinical Pulmonary Medicine, 2011,16(9):1403–1405.
399. Y Xing, G Chen, Y Wang. Analysis of drug resistance and change of rate of Mycobacterium tuberculosis in Huainan area [J]. Acta Universitatis Medicinalis Nanjing, 2012, 32(08):1164–1167.
400. A Xu, J Li, Z Xu. Drug Resistance in Patients with Pulmonary Tuberculosis at Different Ages in Huizhou City [J]. Practical Journal of Cardiac Cerebral Pneumal and Vascular Disease, 2014, 22(8):42–43.
401. J Li, A Xu, Z Xu. Drug Resistance of Tuberculosis Patients in Huizhou [J]. Practical Journal of Cardiac Cerebral Pneumal and Vascular Disease, 2014, 22(8):39–42.
402. Z Han, X Li, X Leng. Prevalence and risk factors of drug resistant Mycobacterium tuberculosis in Huizhou City [J]. Modern Preventive Medicine, 2015, 42(10):1850–1852.
403. X Li, Z Han, X Ke. Analysis on the screening results of drug-resistant tuberculosis in Huizhou City [J]. Practical Preventive Medicine, 2015, 22(05):527–530.
404. X He. Study on the condition and related factors of active pulmonary tuberculosis [J]. Chinese Community Doctors, 2015, 31(12):157–158.
405. X Zhou. Analysis on drug resistance situation and cultivate of pulmonary tuberculosis in Jilin district [J]. China Practical Medicine, 2012, 07(31):162–163.
406. X Yang, Y Yuan, W Zhang. Drug-resistance of re-treatment smears positive tuberculosis patients in Jilin province [J]. Chinese Journal of Public Health Engineering, 2014, 13(6):429–430.
407. Y Yuan, X Yang, G Yang. Monitor analysis of drug resistance tuberculosis in Province [J]. Chinese Journal of Public Health Engineering, 2015,14(1):70–74.
408. Y Yang, Z Han. Analysis on drug resistance situation and cultivate of pulmonary tuberculosis in Jilin district [J]. China Practical Medicine, 2015, 10(4):246–247.
409. J Hu, X Song, W Xu. Drug resistance analysis of 277 strains of mycobacterium　tuberculosis　in　Jiaxing [J]. Chinese Journal of Preventive Medicine, 2014, 15(6):550–552.
410. Z Pan, X Song, M Zhang. Survey on drug resistance situation of tuberculosis in Jiaxing city during 2004–2008 [J]. Chinese Primary Health Care, 2009, 23(11):63–64.
411. G Xu, H Chen, L Zhao. Drug resistance analysis of 2732 strains of acid fast in Jiangmen city [J]. Guangdong Medical Journal, 2015, 36(04):599–601.
412. J Chen, X Zhang. Monitor result analysis of drug resistance tuberculosis during 2000–2004 in Jiangmen city [J]. Journal of Practical Medical Techniques, 2005, 12(9A):2367–2368.
413. J Chen, X Zhang. Monitor result analysis of drug resistance tuberculosis during 2000–2004 in Jiangmen city [J]. Chinese Community Doctors, 2012, 14(16):34–35.
414. Y Shao, H Song, G Li. Epidemiology of drug-resistant Mycobacterium tuberculosis strains circulating in Jiangsu Province in 2010[J]. Acta Universitatis Medicinalis Nanjing, 2013, 33(02):282–285.
415. Du X, Zhu M. Survey and analysis of drug resistance situation of tuberculosis in Changzhou city, Jiangsu province in 2013[J]. Journal of Practical Medical Techniques, 2014, 21(11):1171–1173.
416. D Yang, Y Shao, H Yu. Epidemiology of anti-tuberculosis drug resistance in Jiangsu province [J]. Acta Universitatis Medicinalis Nanjing, 2011, 31(7):1007–1010.
417. W Lu, Y Zhou, C Chen. Prevalence and risk factors for drug resistance tuberculosis in Jiangsu Province: a population based study [J]. 2013, 17(07):560–563.
418. L Qiu, X Jin, Q Huang. Analysis of TB drug resistance investigation in some areas of Jianxi [J]. Modern Preventive Medicine, 2012,39 (2):439–441
419. Q Wang, D Wang, g Y Din. The Effect of Initial Drug Resistance on Treatment Response and acquired drug resistance during standardized Short-Course Chemotherapy for tuberculosis [J]. Anhui Medical Journal, 2013, 34(6):687–689.
420. A Xierzati, H Jianaer. Bacterial culture and drug resistance test of 127 cases of tuberculosis [J]. Modern Chinese Women 2013(9):159–159.
421. Y Su, Y Liu, Y Ma. Analysis of drug sensitive test result of tuberculosis patients [J]. Journal of Diseases Monitor & Control, 2013, 7(3):174–174.
422. D Du, J Jiang, Z Liu. Tuberculosis drug resistance and treatment analysis in Hubei Yichang [J]. China Medicine, 2012, 07(1):50–52.
423. X Yang, Z Wang, Z Du. Analysis of drug resistance situation of tuberculosis patients [J]. Chinese Journal of Health Laboratory Technology, 2008, 18(11):2426–2426.
424. B Tao. The analysis of the Strain distribution of hospitalized tuberculosis patients and drug resistance results [J]. Journal of Clinical Pulmonary Medicine, 2012, 17(5):855–856.
425. X Liu, H Chen, X Liu. Analysis of drug sensitive test result of mycobacterium tuberculosis [J]. Hebei Medical Journal, 2010, 32(15):1260–1261.
426. X Liang, X Yu, X Zheng. Analysis of drug sensitive test result of 364 strains mycobacterium tuberculosis [J]. Chinese Journal of Misdiagnostics, 2010, 10(10):2408–2409.
427. N Li, Y Wu, Hu X. Evaluation of three methods in the detection of Mycobacterium tuberculosis and analysis of drug resistance in isolates [J]. Experimental & Laboratory Medicine, 2013, 31(3):214–216.
428. Y Liu, S Tang, Q Zhang. Analysis on first-and second line drug resistant Patterns in 518 Mycobacterium tuberculosis strains [J].
429. D Pei, S Wang, X Wang. Analysis of drug resistance of Mycobacterium tuberculosis [J]. Modern Preventive Medicine, 2007, 34(8):1429–1431.
430. X Gong, R Tian, F Fang. Present Situation of Multi-drug Resistant Mycobacterium tuberculosis. Chinese Journal of Nosocomiology, 2010, 20(22):3626–3637.
431. H Chen, Y Jin, T Yu. An Investigation on Multidrug Resistant status and gene detection of Mycobacterium Tuberculosis in Changchun City [J]. Chinese Journal of Laboratory Diagnosis, 2010,14(12):1965–1968.
432. Y Duan, X Cui, Z Zhang. Characteristic analysis on drug resistance of Mycobacterium tuberculosis [J]. Journal of Modern Laboratory Medicine, 2007, 22(03):100–101.
433. L Yin, H Sun, L Shen. Characteristic analysis on drug resistance of Mycobacterium tuberculosis [J]. Hebei Medical Journal, 2011, 33(02):190–191.
434. W Zhu, W Zhou, J Li. clinical study of drug resistance mutation detection of Mycobacterium tuberculosis [J]. Journal of Clinical Medicine in Practice, 2015, 19(03):110–111.
435. Z Li, Y Zhou, K Ji. Analysis of drug resistance of Mycobacterium tuberculosis [J]. China Health Industry, 2014(11):173–173.
436. Sun Y. A Study on the Condition of Drug Susceptibility for Mycobacterium Tuberculosis. Central South University, 2007.
437. S Hou, H Fang, X Song. Monitor of initial drug resistance and cultivate of Mycobacterium tuberculosis [J]. Zhejiang clinical medicine, 2008, 10(04):552–552.
438. J Yang. Monitor of initial drug resistance and cultivate of Mycobacterium tuberculosis [J]. Heilongjiang Medicine, 2014, 38(7):801–801.
439. L Xu, W Luo, Y Hao. Mycobacterium tuberculosis laboratory medicine sensitive analysis[J]. Ningxia Medical Journal, 2005, 27(7):451–453.
440. Z Liu, X Zhang, Y Zhang. The status of drug-resistance on Mycobacterium tuberculosis isolated from Xi’an City, and its correlation with the genotyping of the Beijing family [J]. Chinese Journal of Zoonoses, 2008, 24(05):435–438.
441. H Cao, C Wu, L Mi. Investigation of the drug-resistance of Mycobacterium tuberculosis clinical isolates from Xinjiang [J]. Journal of Pathogen Biology, 2010, 5(04):253–255.
442. W Li. Analysis of drug sensitive test for Mycobacterium tuberculosis [J]. Chinese Journal of Modern Drug Application, 2013, 7(14):183–184.
443. Z Zhang, L Mei. Monitor analysis of initial drug resistance for 375 strains Mycobacterium tuberculosis [J]. Occupation and Health, 2006, 22(16):1276–1277.
444. J Li. Analysis and monitoring of drug resistant tuberculosis [J]. The Chinese and foreign health abstract, 2012, 9(35):57–59.
445. Y Xu, S Zhang, Q Zhang. Analysis on drug resistance situation of 139 strains positive culture tuberculosis [J]. Shanxi Medical Journal, 2014, 43(14):1654–1655.
446. L Yin. Analysis of relevant factor for drug resistance test results of tuberculosis [J]. Healthy People, 2014, 8(7):62–62.
447. J Wei, Q Li, Y Zhang. Dynamic analysis of initial drug resistance for initial smear positive pulmonary tuberculosis in Jinhua city during 2008–2012 [J]. Chinese Rural Health Service Administration, 2013, 33(6):646–648.
448. W Wu, Y Hu, S Zhu. Analysis on influential factors of cure rate of pulmonary tuberculosis and drug resistance [J]. Chinese Preventive Medicine, 2012, 13(5):361–363.
449. W Gong, Y Li, X Chen. Progress analysis of Prevention and control tuberculosis work in Jingzhou city.
450. Z Ming, L Xia, X Peng, et al. Transmission of MDR and XDR tuberculosis in Shanghai, China.[J]. Plos One, 2009, 4(2):e4370-e4370.
451. Q Liu, L Zhu, Y Shao, et al. Rates and risk factors for drug resistance tuberculosis in Northeastern China [J]. Bmc Public Health, 2013, 13(1):1–7.
452. Y Hu, B Mathema, W Wang, et al. Prevalence of multidrug-resistant pulmonary tuberculosis in counties with different duration of DOTS implementation in rural China [J]. Microbial Drug Resistance, 2008, 14(3):227–32.
453. Wang D, Yang C, Kuang T, et al. Prevalence of multidrug and extensively drug-resistant tuberculosis in Beijing, China: a hospital-based retrospective study.[J]. Japanese Journal of Infectious Diseases, 2010, 63(5):368–71.
454. X Li. Population-based surveillance of extensively drug-resistant tuberculosis in Shandong Province, China[C]. The seventh national laboratory medicine of middle-aged and young academic conferences of Chinese medical association. 2012:612–4.
455. Y C Qi, M J Ma, D J Li, et al. Multidrug-Resistant and Extensively Drug-Resistant Tuberculosis in Multi-Ethnic Region, Xinjiang Uygur Autonomous Region, China [J]. Plos One, 2012, 7(2):e32103.
456. Y Zhao, H Li, J Xing, et al. Molecular typing of Mycobacterium tuberculosis isolates circulating in Henan, central China [J]. Experimental & Therapeutic Medicine, 2012, 4(5):949–953.
457. Q Wang, S K Lau, F Liu, et al. Molecular epidemiology and clinical characteristics of drug-resistant Mycobacterium tuberculosis in a tuberculosis referral hospital in China [J]. Plos One, 2014, 9(10):e110209-e110209.
458. H Li, H Y Yang, X G Ma, et al. Molecular epidemiological features of Mycobacterium tuberculosis in an endemic region of Henan Province, China.[J]. International Journal of Tuberculosis & Lung Disease, 2012, 16(8):1097–9.
459. Q Chen, Y Pang, Q Liang, et al. Molecular characteristics of MDR Mycobacterium tuberculosis, strains isolated in Fujian, China [J]. Tuberculosis, 2013, 94(2):159–161.
460. Y Deng, Y Wang, J Wang, et al. Laboratory-based surveillance of extensively drug-resistant tuberculosis, China.[J]. Emerging Infectious Diseases, 2011, 17(3):495–7.
461. L Chen, N Li, M Liu, et al. High prevalence of multidrug-resistant tuberculosis in Zunyi, Guizhou Province of China.[J]. Journal of Antimicrobial Chemotherapy, 2011, 66(10):2435–7.
462. X Yuan, T Zhang, K Kawakami, et al. Genotyping and clinical characteristics of multidrug and extensively drug-resistant tuberculosis in a tertiary care tuberculosis hospital in China [J]. Bmc Infectious Diseases, 2013, 13(1):1–9.
463. J Zhang, L Mi, Y Wang, et al. Genotypes and drug susceptibility of Mycobacterium tuberculosis Isolates in Shihezi, Xinjiang Province, China [J]. Bmc Research Notes, 2011, 5(1):1–7.
464. J Wang, Y Liu, C L Zhang, et al. Genotypes and Characteristics of Clustering and Drug Susceptibility of Mycobacterium tuberculosis Isolates Collected in Heilongjiang Province, China[J]. Journal of Clinical Microbiology, 2011, 49(4):1354–62.
465. L Chen, N Li, Z Liu, et al. Genetic Diversity and Drug Susceptibility of Mycobacterium tuberculosis isolates from Zunyi, One of the Highest-Incidence-Rate Areas in China [J]. Journal of Clinical Microbiology, 2011, 50(3):1043–7.
466. S Tang, Q Zhang, J Yu, et al. Extensively drug-resistant tuberculosis at a tuberculosis specialist hospital in Shanghai, China: clinical characteristics and treatment outcomes.[J]. Scandinavian Journal of Infectious Diseases, 2011, 43(4):280–5.
467. S Yan, D Yang, W Xu, et al. Epidemiology of anti-tuberculosis drug resistance in a Chinese population: current situation and challenges ahead [J]. Bmc Public Health, 2010, 11(1):1–10.
468. X H Wang, A G Ma, X X Han, et al. Correlations between drug resistance of Beijing/W lineage clinical isolates of Mycobacterium tuberculosis and sublineages: a 2009–2013 prospective study in Xinjiang province, China [J]. Medical Science Monitor International Medical Journal of Experimental & Clinical Research, 2015, 21:1313–8.
469. H F Ju, X X Wang, G L Li, et al. Characteristics of Mycobacterium tuberculosis genotype and the relationship between Beijing genotype and drug-resistant phenotypes in Tianjin [J]. Zhonghua liu xing bing xue za zhi, 2011, 32(2):116–9.
470. L Zhang, Y Ye, L Duo, et al. Application of genotype MTBDRplus in rapid detection of the Mycobacterium tuberculosis complex as well as its resistance to isoniazid and rifampin in a high volume laboratory in Southern China.[J]. Molecular Biology Reports, 2011, 38(3):2185–92.
471. C H Liu, H M Li, L Li, et al. Anti-tuberculosis drug resistance patterns and trends in a tuberculosis referral hospital, 1997–2009 [J]. Epidemiology & Infection, 2011, 139(12):1909–1918.
472. N Li, X Liao, L Chen, et al. Antibiotic Susceptibility Patterns of Mycobacterium tuberculosis Isolates from Guizhou Province of China against 13 Antituberculosis Drugs [J]. Microbial Drug Resistance, 2015, 21(3).
473. J Li, Y Y Zhang, X H Gui, et al. Prevalence and risk factors on the resistance related to second-line drugs among multi-drug resistant tuberculosis cases in Shanghai, China [J]. Zhonghua liu xing bing xue za zhi. 2012, 33(33):796–8.
474. W Qi, A D Harries, S G Hinderaker. Performance of culture and drug susceptibility testing in pulmonary tuberculosis patients in northern China [J]. International Journal of Tuberculosis & Lung Disease, 2011, 15(1):137–9.
475. Y Yang, S J Tang, Q Zhang, et al. The changes and the significance of cellular immune function in peripheral blood of patients with multidrug-resistant and extensively drug-resistant pulmonary tuberculosis [J], 2011, 34(2):109–13.
476. Y Hu, S Hoffner, W Jiang, et al. Genetic characterisation of drug-resistant Mycobacterium tuberculosis in rural China: a population-based study [J]. International Journal of Tuberculosis & Lung Disease, 2010, 14(2):210–6.
477. J Li, Y Y Zhang, X H Gui, et al. Prevalence and risk factors on the resistance related to second-line drugs among multi-drug resistant tuberculosis cases in Shanghai, China [J]. Zhonghua liu xing bing xue za zhi.2012, 33(33):796–8.
478. G X He, H Y Wang, W Martien, et al. Multidrug-Resistant Tuberculosis, People’s Republic of China, 2007–2009[J]. Emerging Infectious Diseases, 2011, 17(10):1831–8.
479. F L Huang, J L Jin, S Chen, et al. MTB DR-plus results correlate with treatment outcome in previously treated tuberculosis patients.[J]. International Journal of Tuberculosis & Lung Disease, 2015, 19(3):319–25.
480. M A Aziz, A Wright, A Laszlo, et al. Epidemiology of anti-tuberculosis drug resistance (the Global Project on Anti-tuberculosis Drug Resistance Surveillance): an updated analysis [J]. Lancet, 2006, 368(9553):2142–54.
481. Y W Li, X X Li, H Geng, et al. [Epidemic status of drug-resistant Mycobacterium tuberculosis in Shandong province, China [J]. Chinese journal of tuberculosis and respiratory diseases, 2013, 36(9):667–70.
482. X Wang, Q Fu, Z Li. et al. Drug-resistant tuberculosis in Zhejiang Province, China, 1999–2008.[J]. Emerging Infectious Diseases, 2012, 18(3):496–8.
483. X Shen, K Deriemer, Z A Yuan, et al. Drug-resistant tuberculosis in Shanghai, China, 2000–2006: prevalence, trends and risk factors.[J]. International Journal of Tuberculosis & Lung Disease, 2009, 13(2):253–9.
484. K Xu, S Bi, Z Ji, et al. Distinguishing nontuberculous mycobacteria from multidrug-resistant Mycobacterium tuberculosis, China.[J]. Emerging Infectious Diseases, 2014, 20(6):1060–2.
485. J W Lv, Y Z Yang, Q F Wu. et al. Comparative analysis of 2005,2009 tuberculosis drug surveillance in Shenzhen[J]. Chin J Prim Med Pharm, 2011, 18(13):1741–1742.
486. D Luo. The monitoring of drug resistant of mycobacterium tuberculosis in Shenzhen [J]. Journal of Clinical Pulmonary Medicine, 2006.11(3):304–305.
487. L Xu, Y Yang, Q Wu, et al. Analysis of first line anti-TB drug resistance surveillance in Shenzhen [J]. Chinese Journal of Anti tuberculosis, 2010.

## Abbreviations

CDC: Chinese Center for Disease Control and Prevention
CNKI: China National Knowledge Infrastructure
DOTS: Directly-Observed Treatment Strategy
ESRI: Environmental Systems Research Institute
MDR-TB: Multidrug-resistant tuberculosis
MTB: Mycobacterium tuberculosis
TB: Tuberculosis
WHO: World Health Organization
